# Integrative genomic deconvolution of rheumatoid arthritis GWAS loci into gene and cell type associations

**DOI:** 10.1186/s13059-016-0948-6

**Published:** 2016-04-30

**Authors:** Alice M. Walsh, John W. Whitaker, C. Chris Huang, Yauheniya Cherkas, Sarah L. Lamberth, Carrie Brodmerkel, Mark E. Curran, Radu Dobrin

**Affiliations:** Immunology, Janssen Research and Development, LLC., 1400 McKean Rd., Spring House, PA 19477 USA; Discovery Sciences, Janssen Research and Development, LLC., 3210 Merryfield Row, San Diego, CA 92101 USA

**Keywords:** Genome-wide association studies (GWAS), Epigenomics, Expression quantitative trait loci (eQTLs), Rheumatoid arthritis

## Abstract

**Background:**

Although genome-wide association studies (GWAS) have identified over 100 genetic loci associated with rheumatoid arthritis (RA), our ability to translate these results into disease understanding and novel therapeutics is limited. Most RA GWAS loci reside outside of protein-coding regions and likely affect distal transcriptional enhancers. Furthermore, GWAS do not identify the cell types where the associated causal gene functions. Thus, mapping the transcriptional regulatory roles of GWAS hits and the relevant cell types will lead to better understanding of RA pathogenesis.

**Results:**

We combine the whole-genome sequences and blood transcription profiles of 377 RA patients and identify over 6000 unique genes with expression quantitative trait loci (eQTLs). We demonstrate the quality of the identified eQTLs through comparison to non-RA individuals. We integrate the eQTLs with immune cell epigenome maps, RA GWAS risk loci, and adjustment for linkage disequilibrium to propose target genes of immune cell enhancers that overlap RA risk loci. We examine 20 immune cell epigenomes and perform a focused analysis on primary monocytes, B cells, and T cells.

**Conclusions:**

We highlight cell-specific gene associations with relevance to RA pathogenesis including the identification of FCGR2B in B cells as possessing both intragenic and enhancer regulatory GWAS hits. We show that our RA patient cohort derived eQTL network is more informative for studying RA than that from a healthy cohort. While not experimentally validated here, the reported eQTLs and cell type-specific RA risk associations can prioritize future experiments with the goal of elucidating the regulatory mechanisms behind genetic risk associations.

**Electronic supplementary material:**

The online version of this article (doi:10.1186/s13059-016-0948-6) contains supplementary material, which is available to authorized users.

## Background

Rheumatoid arthritis (RA) is a common autoimmune disease that results in progressive disability. RA primarily affects the small joints of the hands and feet, where immune cells invade the lining of the joint, causing synovial inflammation and hyperplasia. Disease progression leads to cartilage and bone destruction as well as systemic comorbidities that result in higher mortality rates in RA patients than healthy adults [1]. The genetic factors underlying susceptibility to RA have been examined with multiple genome-wide association studies (GWAS) that have identified over 100 single nucleotide polymorphisms (SNPs) associated with RA [2]. However, the translation of these findings into disease understanding and therapeutic interventions is not straightforward. Besides the difficulty in identifying disease-casual variants (and not those in high linkage disequilibrium [LD] with the true causal variant), most reported GWAS SNPs do not reside in protein-coding regions and may be near many candidate genes. These challenges have limited the ability to translate genetic studies into RA disease understanding.

While most identified disease-associated genetic variants do not result in functional mutations, multiple lines of evidence support a gene regulatory role for these variants. It has been demonstrated that non-coding GWAS SNPs are more likely to reside in DNaseI hypersensitivity sites indicative of *cis*-regulatory elements [3]. Moreover, immune disease-associated SNPs are highly enriched at intergenic regulatory enhancers, with a limited set perturbing known transcription factor motifs [3–7]. There are also several mechanistic demonstrations of specific GWAS-identified SNPs that have been linked to disease through gene mis-regulation [8, 9]. Together, these studies provide strong evidence that SNPs identified through GWAS need to be further annotated with additional data to understand their function.

The regulatory role of GWAS loci is further supported by the finding that GWAS SNPs are enriched at DNA variants correlated with gene expression changes, known as expression quantitative trait loci (eQTLs) [10]. eQTLs are a powerful tool to connect SNPs of unknown function with expression of putative disease-relevant genes. Toward the goal of elucidating the gene regulatory role of disease-associated variants, previous studies have used publicly available eQTL datasets to annotate GWAS SNPs [2, 11, 12]. Increasingly, eQTLs mapped from sorted cell populations or tissues are used because of evidence that gene regulation is highly context specific [4, 13–15]. It is often prohibitively difficult to obtain eQTL datasets from large patient cohorts or access disease-relevant cells or tissues; therefore, data from healthy cohorts and not the disease of interest are used. However, we hypothesize that studies of relevant patient populations could provide better information than these non-disease datasets.

Herein, we present the results of an investigation to address some of the challenges described above. We mapped eQTLs using whole-genome sequencing data and whole blood gene expression data from a population of 377 unrelated RA patients. To our knowledge, this is the first study to map eQTLs from RA patient samples. We find greater than 6000 genes with significant eQTLs that are enriched with RA relevant pathways. The RA mapped eQTLs are enriched at RA GWAS loci and enhancers from immune cell epigenome maps. Comparison to eQTLs from healthy donors revealed greater enrichment in RA GWAS loci in our dataset derived from RA patients. Next, we associated known RA GWAS loci with proposed gene and cell type annotations by performing an integrative genomics analysis of: RA eQTLs, RA GWAS loci, and immune cell epigenome maps. We find greater cell type-specific chromatin activity at enhancers overlapping RA GWAS loci compared to gene body/promoters overlapping GWAS loci across various cell type datasets. As our deconvolution converts loci associations into cell type and gene associations, it simplifies the choice of an experimental system for validation. As an example, we identify *FCGR2B* in B cells as possessing both intragenic and enhancer regulatory GWAS hits, suggesting that this gene is potentially a key RA driver in B cells. We provide our results as a foundation to generate hypotheses for the design of validation experiments, which could tease apart the genetic and biologic mechanisms underlying the development and progression of RA, a disease where there remains a large unmet therapeutic need.

## Results

### eQTL mapping from a RA cohort

Gene expression and genotype data were combined to map eQTLs from a population of 377 RA patients with moderate to severe disease and inadequate response to methotrexate [16]. A summary of patients used in this study is given in Table 1. Transcriptome data (Affymetrix microarray) from whole blood were compared to matching genotypes generated from whole-genome sequencing [17]. In brief, eQTL mapping was performed by linear regression on adjusted data and false discovery rate (FDR) was estimated with a permutation method separately for local (*cis* defined as less than 1 Mb distance from SNP to gene) and distant associations (*trans*, greater than 1 Mb from SNP to gene including across chromosomes; see “Methods”).

**Table 1:** Summary statistics on patient population

Age (years)	Min	18
	Mean	52
	Max	83
Gender	Male	81 (21.5 %)
	Female	296 (78.5 %)
Disease duration (years)	Min	0.3
	Mean	7.3
	Max	39.7
DAS28-CRP score	Min	3.6
	Mean	5.9
	Max	7.9

**Fig. 1:**
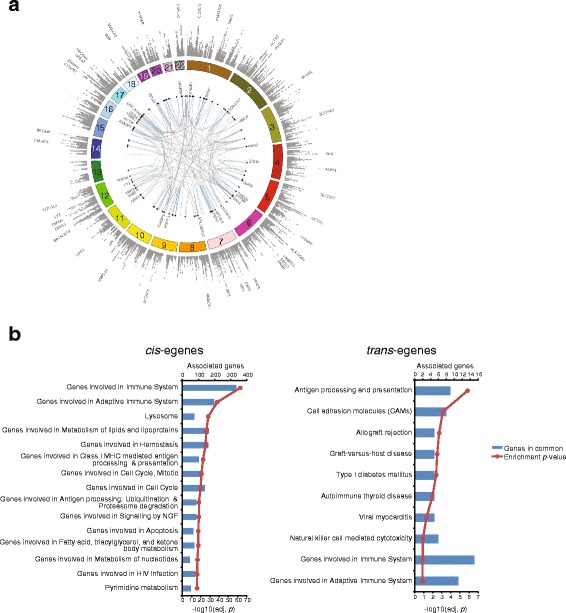
Overview of eQTLs from the blood of RA subjects and canonical pathway enrichment of genes with eQTLs. **a** *Circos plot* for eQTLs mapped in whole blood from RA subjects. Outermost track of *gray dots* shows a Manhattan plot of *cis*-eQTLs (FDR < 5 %) with egenes with lowest *p* values labeled. The *inner links* connect *trans*-eQTLs to the associated egene (only *trans*-eQTLs with nominal *p* values <10^−14^ are shown for clarity). *Blue links* represent associations within the same chromosome whereas *gray links* represent associations that span across chromosomes. **b** egenes associated with eQTLs in RA whole blood samples were tested for enrichment with the MSigDB term database separately for egenes associated in *cis* (*left*) or in *trans* (*right*)

We identified *cis*-eQTLs corresponding to 6194 unique genes (referred to as egenes) at the 5 % FDR level (described in Fig. 1a, Table 2, Additional file 1 for the complete set of connections and Additional file 2 for the list of all SNPs tested but not necessarily found to be associated with gene expression). An additional 236 unique egenes were mapped to *trans* loci (5 % FDR; Additional file 3). In order to identify the biological processes represented in this set of genes, we performed enrichment analysis using knowledge-based canonical pathways (see “Methods”). The top associated pathways were genes involved in the immune system and the adaptive immune system. Other associated terms were biological processes with known importance in RA such as antigen processing and presentation and pathways such as lipid metabolism and cell cycle (Fig. 1b). Genes with *trans*-eQTLs were enriched for pathways related to immune regulation with the top enrichment for antigen processing and presentation. The pathway enrichment for *trans*-eQTL egenes was largely driven by HLA genes (e.g. HLA-DRB4, HLA-C, HLA-B, HLA-DPA1, and HLA-DQA1 appear in the gene sets for graft-versus-host disease, allograft rejection, and cell adhesion molecules). This is in agreement with previous studies that found ~50 % of *trans*-eQTLs from peripheral blood map within the HLA region [18].

**Table 2:** Summary of eQTLs detected in whole blood of RA patients

	Unique eSNPs	Unique egenes	FDR
*Cis*	712,539	6194	0.05
*Trans*	24,316	236	0.05

### Enrichment of RA eQTLs in the GWAS catalog and comparison to other published eQTL studies

**Fig. 2:**
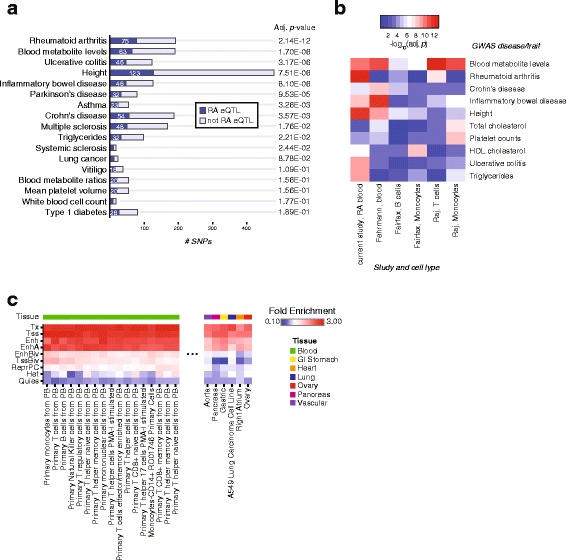
Comparison of RA eQTLs to published GWAS catalog and fold-enrichment of RA eQTLs in chromatin states from diverse tissues. **a** Comparison of RA *cis*-eQTLs to reported GWAS SNPs in the NHGRI catalog. The top 17 diseases or traits are shown, ranked by enrichment *p* value from one-sided Fisher’s exact test. The number of GWAS reported SNPs overlapping with RA eQTLs in our dataset are shown for each disease or trait. **b** Comparison of enrichment of GWAS SNPs in several eQTL studies. The top ten GWAS diseases or traits are shown. The eQTL studies are labeled by their first author and cell type or tissue where gene expression was measured. Only *cis*-eQTLs were considered. **c** Enrichment of RA eQTLs in chromatin states. Both *cis*- and *trans*-eQTLs were compared to positions of the following chromatin states in diverse tissues: transcription start sites (*TSS*), actively transcribed (*Tx*), enhancers (*Enh*), active enhancers (*EnhA*), heterochromatin (*Het*), bivalent enhancers (*EnhBiv*), bivalent/poised TSS (*TssBiv*), repressed Polycomb (*ReprPC*), and quiescent/low (*Quies*) regions. Only a subset of the cell or tissue types with the highest or lowest fold-enrichment in enhancer states are shown to ease representation. The full results are shown in Additional file 5: Figure S2

Previous studies have found that GWAS SNPs are enriched in eQTLs [10]. In order to determine the overlap of disease-associated SNPs with eQTLs in our study, we compared the identified RA eQTLs with the NHGRI catalog of published GWAS [19]. The diseases or traits with the most significant overlap with RA *cis*-eQTLs are shown in Fig. 2a. Of the 184 variants reported to be associated with RA susceptibility, 75 were associated with gene expression in *cis* in our analysis. There were 69 unique genes mapped to these eQTLs. When considering SNPs in high LD (*r*^2^ ≥ 0.8) with the GWAS reported SNPs, 82 reported RA GWAS SNPs were associated with 80 unique genes in *cis* in our analysis. The RA eQTLs were also enriched for other known autoimmune disease GWAS, including ulcerative colitis, Crohn’s disease, and multiple sclerosis. The RA *cis*-eQTLs detected in this study were compared to *cis*-eQTLs from previously published studies, including those mapped from sorted populations of B cells, T cells, and monocytes from healthy donors [13, 14, 18] (Additional file 4: Figure S1). We repeated the comparison of *cis*-eQTLs to GWAS loci in these non-RA populations (Fig. 2b). The enrichment with RA GWAS SNPs was highest in our RA whole blood study; with some overlap also present in the Raj et al. study of T cells [14]. This demonstrates that eQTL studies of RA patients may yield improved power to discover potential disease-regulatory genes compared to studies using non-RA donors. The *cis*-eQTLs from Fehrmann et al. are enriched in inflammatory bowel disease GWAS SNPs, which is in agreement with the fact that the subjects in that study included 49 individuals with inflammatory bowel disease [18].

### Comparison of RA eQTLs with epigenomics datasets

Genetic variants associated with immune diseases have been shown to overlap distal regulatory enhancer elements [4]. Based on this finding, we sought to compare the location of the RA eQTLs with enhancer elements (derived from histone modification epigenome studies) from cell or tissue specific datasets. We expect that when eQTLs and epigenomes are compared between cell types, both cell type-specific elements and common elements will be found. This expectation is based on previously published analysis indicating that more than 50 % of eQTLs are shared when comparing tissues [15] and data from the Roadmap Epigenomics Project, which identified 2.3 million enhancers across 111 epigenomes, of which 57,840 formed a housekeeping cluster that was active across all lineages [5]. Nonetheless, we sought to confirm that RA whole blood eQTLs were enriched in peripheral immune cell types. We used annotated chromatin states from 97 cell or tissue-specific datasets from the Roadmap Epigenomics project and ENCODE [5, 20] generated with ChromHMM [21] (see “Methods”). The eQTLs were enriched at actively transcribed regions and active transcription start sites (TSS) in all cell types but exhibited greater fold-enrichment in the enhancers and active enhancers of primary cell types from peripheral blood (Fig. 2c; Additional file 5: Figure S2). The greater specificity of enrichment of RA eQTLs at enhancer elements than promoters may result from enhancer activity being more cell type-specific than promoter activity [5, 22].

Analysis based on a local permutation method demonstrated that the enrichment of RA eQTLs in enhancer elements was statistically significant [23] (see “Methods” and Additional file 5: Figure S2B, C).

### Identification of RA therapeutic targets in monocytes, B cells, and T cells

**Fig. 3:**
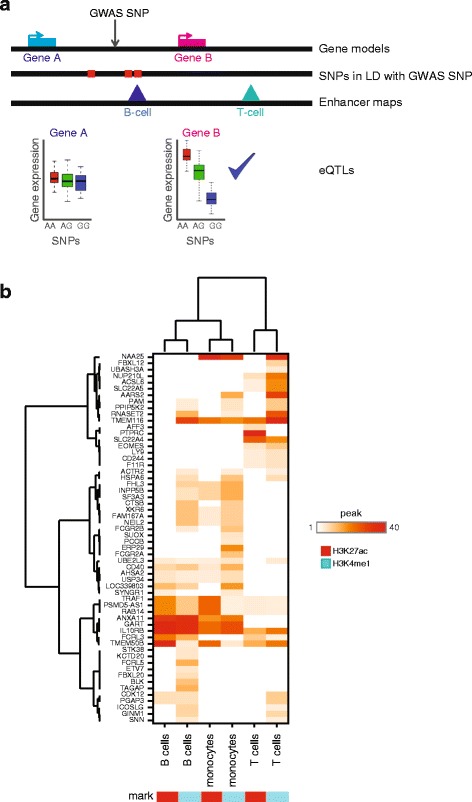
Integration of epigenomics datasets allows cell-type annotation for RA GWAS and RA eQTL connections. **a** *Schematic* demonstrating the approach to connect reported GWAS SNPs of unknown function to putative causal genes using epigenomics and eQTL datasets. **b** *Heatmap* of genes identified to be associated with RA GWAS SNPs in primary monocyte, T cell, and B cell datasets

RA GWAS variants at enhancers likely function by causing dysregulation of their target genes; therefore, these genes may represent novel therapeutic targets or pathways for the treatment of RA. Having confirmed that RA blood eQTLs are enriched at immune cell enhancers and RA GWAS loci, we combined the datasets to identify the potential target genes of RA GWAS loci that overlap immune cell enhancers. By integrating RA blood eQTLs, RA GWAS, and immune cell enhancer datasets, we identified triple hit regulatory regions of the genome and their potential target genes. Our approach addresses three major problems with the interpretation of RA GWAS loci: LD between SNPs, a lack of cell type-specific information, and that the target genes of putative regulatory SNPs are unknown. To overcome these challenges, we developed an integrative analysis methodology that takes all SNPs in LD with the RA GWAS hits, identifies those that overlap blood cell type enhancers, and then uses eQTLs to link these to genes that possess active chromatin marks in the corresponding cell type (Fig. 3a). Through this approach we annotate intergenic GWAS hits with a cell type of activity and target gene(s).

In brief, we started by identifying enhancer regions using ChIP-seq maps of histone H3 that are modified with acetylation of lysine 27 (H3K27ac) and mono-methylation of lysine 4 (H3K4me1). Then we identified RA GWAS SNPs from Okada et al. [2] and SNPs in high LD (*r*^2^ ≥ 0.8) with the RA GWAS SNPs and overlapped these with the enhancer regions (HLA-region SNPs on chromosome 6 were excluded from this analysis and will be treated separately). Active genes were identified by looking for active promoter marks, H3K27ac or tri-methylation of lysine 4 (H3K4me3), at gene TSS. Next, enhancers overlapping RA GWAS loci were linked to target genes using eQTLs between the active genes and the SNPs overlapping the enhancers (see “Methods” for complete description). We focused our interpretation on primary monocytes, primary B cells, and primary T cells from peripheral blood (Fig. 3b) and we provide the complete results for all cell types in Additional file 6. While monocytes are not directly involved in RA disease, they differentiate into other cell types that are involved in disease, such as dendritic cells and macrophages. H3K4me1 peaks identified more RA GWAS/eQTL overlapping enhancers than H3K27ac, which may be reflective of the higher genome coverage of H3K4me1. We plotted H3K4me1 connections in all available T cell datasets and found that 69 % of genes were identified in multiple T cell types (Additional file 7: Figure S3) suggesting the majority of H3K4me1 connections do not result from the chance placement of H3K4me1. Previous publications have suggested that enhancers possessing H3K27ac are likely active in that lineage [24, 25], whereas enhancers possessing only H3K4me1 are likely to represent enhancers that are primed to become activated upon stimulation, in sub-lineages, or in disease [4, 26, 27]. Hierarchical clustering demonstrated that the three cell types had distinct patterns of SNP-enhancer-gene connections. Interestingly, B cells and monocytes were more similar than B cells and T cells, potentially reflecting shared roles in antigen presentation during RA versus genes shared from common progenitors.

Our methodology prioritized testable connections between GWAS loci at enhancers, target genes, and the cell types in which the GWAS regulate expression. Of the 58 genes that had *cis*-eQTLs overlapping RA GWAS and enhancer regions (either H3K27ac or H3K4me1 marks), 12 genes were associated with all three cell types. These genes were *ACTR2*, *HSPA6*, *IL10RB*, *PAM*, *PPIP5K2, PSMD5-AS1*, *RAB14*, *RNASET2*, *TMEM50B*, *TMEM116*, *TRAF1*, and *UBE2L3*. There were other genes, such as *FCRL5* and *BLK*, that had *cis*-eQTLs overlapping enhancer regions and GWAS loci in B cells, but not monocytes and T cells. Genes with monocyte-specific *cis*-eQTLs included *SUOX* and *PCCB.* Genes with RA GWAS overlapping enhancers in T cells only included *IL2* and *LY9*.

The above methodology identified genes likely associated with intergenic RA GWAS. However, we also wanted to consider RA GWAS loci that overlap genes or their promoters in the immune cell types considered. We identified 68 genes that had RA GWAS loci overlapping their promoter or any part of their transcript and were considered “active” in the immune cell epigenome maps (possessed active chromatin marks at their promoters; see “Methods” and Additional file 8: Figure S4). The majority of these genes (51 of 68 genes) are active in all three cell-specific datasets (e.g. *GATA3* and *IL6R*), which is reflective of the lower cell-type specificity of promoters [5, 22]. However, some genes are specific to certain cell types, such as *PADI4*, which overlaps a RA-associated SNP, and only possess active chromatin marks at its promoter in monocytes (Additional file 9: Figure S5). It was also tested whether H3K4me3 outside of gene promoter regions near TSSs would be useful to provide additional connections between GWAS loci and putative causal genes. We found that analysis of H3K4me3 peaks complemented the analysis described here and did not point to any additional genes that could be associated with GWAS loci.

Separately from the *cis*-eQTLs described in detail above, we also queried whether there were any RA GWAS SNPs associated with gene expression of distal genes (*trans-*eQTLs). We identified *trans*-eQTLs corresponding to three unique egenes that overlapped reported RA GWAS SNPs (or those in high LD with the reported SNPs). These *trans*-eQTLs were all located in the HLA region on chromosome 6 and were associated with expression of *SAMD14*, *BICD1*, and *PTCRA. SAMD14* encodes for Sterile Alpha Motif Domain Containing 14, a protein without known function or relation to autoimmunity. *BICD1* encodes for Bicaudal D Homolog 1, a protein involved in golgi-endoplasmic reticulum transport. BICD1 is known to interact with STAT3, GSK3B, PLK1, and MAPK14, proteins involved in immune cell signaling. *PTCRA* encodes for Pre T-Cell Antigen Receptor Alpha, which forms part of the pre-T-cell receptor complex and regulates T cell development. The *trans*-eQTLs for these genes are visualized in Additional file 10: Figure S6. These genes have not, to our knowledge, previously been documented as associated with any RA GWAS loci.

### Disease-relevance of RA GWAS-associated genes

**Fig. 4:**
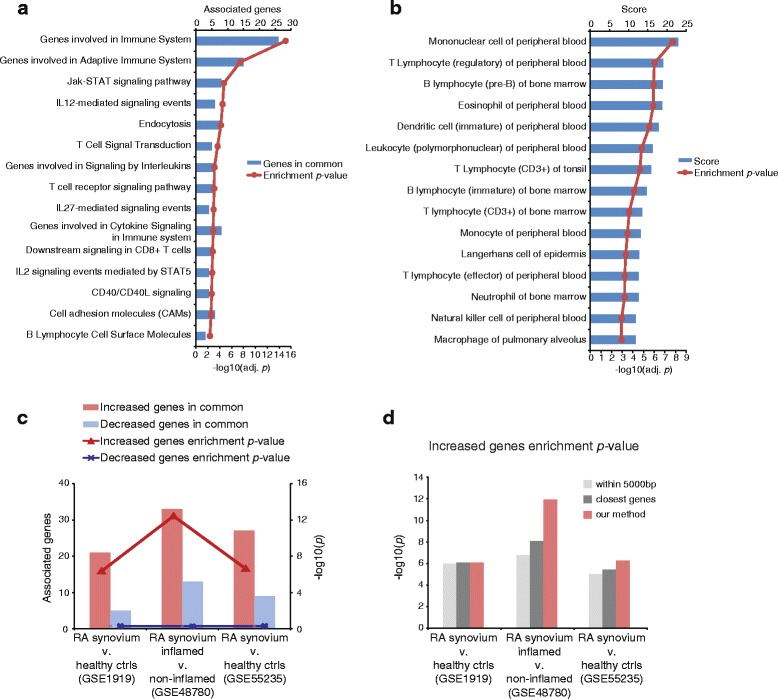
Canonical pathway and cell-type enrichment of identified RA GWAS-associated genes and enrichment with RA gene expression disease profiles. Genes identified as associated with RA GWAS SNPs in monocytes, B cells, or T cells were tested for enrichment with the MSigDB term database **a** or a database of cell type-specific gene expression **b**. **c** The genes identified were compared to genes differentially expressed in three publicly available RA synovial tissue datasets with the GEO IDs indicated. The enrichment *p* value is the unadjusted *p* value from one-sided Fisher’s exact test. **d** The enrichment with the increased genes in the same RA synovial tissue datasets were compared with gene lists generated by choosing the closest genes to the RA GWAS SNPs (“closest genes”; the same number of genes are selected per SNP as with our method) or choosing any gene(s) within 5000 bp of the GWAS SNP (“within 5000 bp”)

We tested whether the genes associated with RA GWAS SNPs (not including MHC class II risk SNPs) were enriched for genes related to RA pathogenesis. We tested the set of genes described above derived using epigenome maps from peripheral B cells, T cells, and monocytes. The immune cell RA-associated genes were enriched for general immune system pathways and more specific pathway terms such as JAK-STAT signaling pathway, IL-12-mediated signaling events, and T cell receptor signaling pathway (Fig. 4a). These RA-associated genes were also enriched for genes expressed in certain peripheral blood cell types. The greatest enrichment was observed for genes associated with mononuclear cell of peripheral blood, T lymphocyte (regulatory) of peripheral blood, and B lymphocyte (pre-B) of bone marrow (Fig. 4b). Because there were several RA GWAS SNPs that were not annotated with a gene in the chosen peripheral blood cell types by our approach, we expanded our set of genes to include genes that had been previously been associated with these reported risk SNPs. The results of this analysis are shown in Additional file 11: Figure S7.

We next tested whether the genes we identified were enriched in genes identified as differentially expressed in RA datasets. Unlike the use of knowledge-based gene sets, this allows for the inclusion of genes that may be important for disease but are not yet well established in the literature and absent from pathway databases. The immune cell RA-associated genes we identified were enriched for genes increased in RA synovial tissue compared to normal tissue or in inflamed compared to non-inflamed synovial tissue (Fig. 4c). Between 21 and 33 RA GWAS-associated genes were significantly increased in these datasets. A total of 68 out of 120 RA GWAS-associated genes were differentially expressed in at least one of these datasets. We compared these results to gene sets derived from other automated methodologies to annotate GWAS SNPs with genes. First, a gene set was generated from the same set of GWAS SNPs annotated by our method, by simply choosing the closest gene(s) to the reported GWAS SNP using the same number of genes per SNP (“closest genes”). A second gene set was generated by choosing any genes within 5000 bp of the reported SNP (“within 5000 bp”). We observed that our method selected more genes that were differentially expressed in the orthogonal disease datasets, resulting in lower enrichment *p* values (Fig. 4d). Overall, this analysis suggests that we identified genes expressed in immune cell types that are increased in inflamed synovial tissue of RA patients and that our method can nominate more disease-relevant genes than previous approaches that do not incorporate eQTL and epigenome datasets.

In addition to this traditional enrichment analysis, the RA-associated genes were analyzed with a network-based methodology to assess the network connectivity and identify biologically relevant associated genes (Additional file 12: Figure S8). It has previously been shown that disease associated genes cluster together on gene interaction networks [28–31]. For our comparison, we compared the genes identified in Fig. 4 (as well as MHC class II risk genes) to genes from the NCBI Phenotype-Genotype Integrator for the trait “Arthritis, Rheumatoid” (http://www.ncbi.nlm.nih.gov/gap/phegeni). A publicly available human interactome was utilized [28]. We found that, indeed, the GWAS-associated genes we identified formed a connected component with 16 members (and a second large component of nine genes) compared to ten directly connected genes for the NCBI annotated gene list (corresponding to a z-score based on 100,000 permutations of 4.6 and 2.7, respectively). The GWAS-associated genes we identified have a mean shortest distance of 1.78 versus 1.90 for the NCBI list.

## Discussion

### Mapping gene regulatory components of RA

Understanding the genetic and regulatory component of complex diseases such as RA remains a large challenge. Here, we present a resource to study the connections between RA risk loci and gene expression. We used matched whole-genome sequencing and mRNA profiling from 377 patients with moderate to severe RA. This dataset has the advantage of being disease-specific, unlike eQTLs derived from healthy (non-RA) subjects. We demonstrated that the eQTLs mapped from the whole blood of RA patients are more enriched for RA GWAS SNPs than other eQTL datasets (Fig. 2). We confirmed that eQTLs mapped from RA patients are enriched in enhancer regions of relevant cell types such as peripheral blood B cells and T cells. We mapped previously reported RA GWAS loci to enhancers from peripheral blood datasets and then genes using our eQTLs. This method was able to identify connections between genes and regions associated with RA risk, providing a mechanism to better understand RA disease biology as well as potential pathways for drug discovery.

The finding that our eQTL dataset mapped from RA patients is more enriched with RA GWAS loci that previously published datasets in non-RA cohorts could be due to several factors, which are explored further in Additional file 13: Figure S9. One possible explanation is that the eQTL variants have higher allele frequencies in RA subjects, therefore boosting the power to detect associations with gene expression. However, GWA studies indicate that the allele frequencies should not be greatly different for most variants in RA cases versus healthy controls. Therefore, this is unlikely to explain the observed difference. We confirmed this by comparing the allele frequencies of eQTLs observed in our cohort to the allele frequencies for the same variants in the 1000 genomes EUR population (Additional file 13: Figure S9A–C). A second possible explanation for the enrichment of RA GWAS loci in our RA dataset is that RA blood contains a larger proportion of relevant cell types compared to healthy blood, which could boost the power to detect eQTLs for genes expressed predominantly in these cell types. Indeed, there are several publications documenting changes in cell frequency in peripheral blood of RA patients compared to non-RA healthy controls [32–34]. A comparison of egenes from our study to those from healthy donors demonstrated that egenes detected uniquely in RA blood (and not healthy donors) were highly expressed in relevant cell populations such as T lymphocytes (Additional file 13: Figure S9F). A third explanation for the difference between RA and healthy eQTLs, which is potentially the most intriguing, is that the conditions present in RA patients result in context-specific gene regulation resulting in detection of eQTLs not present in healthy donors. In support of this hypothesis, stimulation-dependent eQTLs have been reported in human monocytes stimulated with lipopolysaccharide or interferon-ɣ [35]. In order to further explore this possibility for the eQTLs detected here, further focused studies would need to be performed.

### Identification of novel disease-relevant genes

While many of the genes we identified have been mapped to RA GWAS loci in previous reports and have documented associations with RA, such as *PTPN22* (protein tyrosine phosphatase, non-receptor type 22) [36], others are less well studied, such as *PAM* (Peptidylglycine alpha-amidating monooxygenase), an enzyme that catalyzed the C-terminal amidation of peptides. Another example of a gene not previously linked to RA genetics is *CTSB* (Cathepsin B), a proteinase involved in amyloid precursor protein processing that is known to be elevated in the synovial fluid of RA patients and could be involved in collagen destruction [37].

**Fig. 5:**
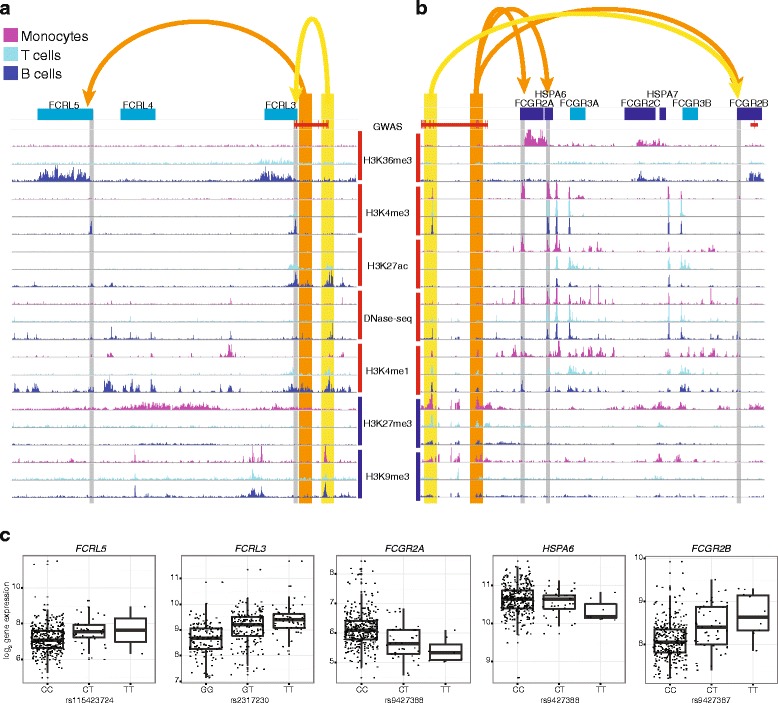
Example RA risk enhancers and their target genes. **a** A genome *browser view* showing a 250 kp window around the RA risk loci rs2317230. The RA GWAS hit and SNPs in LD are shown in *red* and overlap two immune enhancer regions. *Yellow* and *orange* are used to highlight enhancer locations and point to their target genes whose active TSSs are highlighted with *gray*. Histone modification tracks show the fold change between ChIP and control read counts. Active histone modifications have *red bars* next to their labels while repressive marks have *blue*. Genes on the positive and negative strands are shown in *blue* and *cyan*, respectively. **b** A genome *browser view* is shown as in **a**, but around the RA risk loci rs72717009. **c** Gene expression (normalized log2-transformed values) is plotted versus the genotype at representative enhancer-localized SNPs for the same genes shown in **a** and **b**

Our approach facilitates the interpretation of GWAS loci in specific cellular contexts, suggesting an experimental cell type that could be used for validation of proposed therapeutic targets or disease-specific biomarkers. As an illustrative example, *FCRL5* (Fc receptor-like 5) was identified as associated with rs2317230 on chromosome 1 and overlapping a B cell-specific enhancer (Fig. 5a, c). Recent literature reports support our cell-type association for *FCRL5. FCRL5* is known to be involved in B cell signaling and is highly expressed in tonsil plasma cells, naïve B cells, and memory B cells [38]. The FCRL5 cytoplasmic domain has both an immunoreceptor tyrosine-based activation motif (ITAM)-like sequence and two consensus immunoreceptor tyrosine-based inhibitory motifs (ITIM) and appears to have an inhibitory effect on BCR signaling [39]. FCRL5/IgG interaction results in IgG auto-regulation, although FCRL5 does not bind IgG in a traditional manner via the Fc like Fc receptors [40]. Furthermore, in the treatment of RA with Rituxan (anti-CD20), low *FCRL5* mRNA levels in B cells from whole blood have been shown to predict positive response [41]. Taken together, there is strong evidence to support the genetic association with *FCRL5* in the B cell lineage. In contrast, *FCRL3* is associated RA in both T cells and B cells but is repressed by H3K27me3 in monocytes (Fig. 5a, c).

*FCGR2B* is an example of a gene that overlaps a GWAS SNP and also has a *cis*-eQTL that overlaps a different reported GWAS SNP. A *cis*-eQTL for *FCGR2B* overlaps with enhancers in monocytes and B cells and the reported risk SNP rs72717009 (OR 1.13; [2]). A different reported risk SNP at chr1:161644258 (OR 1.15; [2]) also overlaps with the *FCGR2B* transcript (Fig. 5b, c). The two independent GWAS associations between RA and *FCGR2B* highlight its potential importance. Literature supports the association between RA and FCGR2B expression in B cells as *FCGR2B* expression is reduced in memory B cells and plasmablasts from RA patients compared to healthy controls and FCGR2B expression is associated with levels of anti-citrullinated autoantibodies [42].

*TAGAP* (T-cell activation Rho GTPase activating protein) is another example gene that was also identified with a *cis*-eQTL overlapping a B cell-specific enhancer, but a specific role for *TAGAP* in RA disease has not previously been demonstrated. Our study suggests that the function could be specific to B cells. This is further supported by gene expression data that demonstrate that *TAGAP* expression is highest in naïve B cells compared to CD4-positive and CD8-positive T cell subtypes or other B cell subtypes [43]. Furthermore, a previous publication found that the association of a TAGAP variant (rs182429) was stronger in patients with anti-cyclic citrillinated peptide (anti-CCP) antibodies [44 ].

### Limitations of the current approach

While our study was able to annotate many reported RA GWAS SNPs with potential genes, there were several SNPs that were not associated with enhancers or located within any active genes in the blood cell types evaluated. One possibility is that the medications used by the subjects in this study altered gene transcription in the blood and either masked some true effects or created false positives. The cohort used here was relatively homogeneous in current medications because this was an inclusion criterion of the study [16]. All subjects were on a stable dose of methotrexate with no exposure to biologics such as TNF-alpha inhibitors. A second possibility is that these GWAS SNPs are associated with regulation of genes expressed in other cell types. The known pathology of RA suggests these SNPs could function in fibroblast-like synoviocytes (FLS), as it is known that the FLS in affected joints of individuals with RA exhibit a transformed, invasive phenotype [45]. Additionally, genome-wide analysis of DNA methylation has identified a stable RA-specific signature at disease-related genes [46–48]. In these studies, several of the SNPs that were not annotated in blood cell types, were previously described in analyses of RA FLS [49]. For example, the reported risk SNP rs10175798 was not annotated by any gene in our study, but is proximal to *LBH*. This gene was found to be differentially expressed and differentially methylated in RA FLS [49]. Furthermore, *LBH* was shown to regulate proliferation in FLS [50]. Unfortunately, FLS could not be included in our analysis as there is currently no histone modification or eQTL data available in this cell type. To date, the large number of RA FLS samples needed for eQTL mapping has been difficult to obtain as synovial biopsies are not routinely performed. However, new methods to improve synovial biopsy collection may make this type of study possible in the near future [51].

Besides FLS, our study may also not be able to detect eQTLs from cell types that are rare in whole blood. We focused our analysis on epigenome maps from monocytes, B cells, and T cells, but a focused study using isolated regulatory T cells or other cell types of interest will likely be necessary to fully annotate the RA risk loci with prioritized gene connections. In our present study, we use co-overlap of GWAS SNP and eQTL SNP with histone modification ChIP-seq peaks to link GWAS loci to target genes without assessing the exact colocalization of the two signals at one SNP. The two pieces of information are used distinctly: (1) the RA GWAS hit is used to associate the enhancer’s dysregulation with RA; and (2) the eQTL is used to link the enhancer to target genes. Further insights into RA genetics would be obtained through careful measurement and testing of eQTL and GWAS signal colocalization; however, such studies would require larger samples sizes and eQTLs from a wide variety of purified cell types in resting, active, healthy, and diseased states [52].

Additional limitations of the present study are the availability of epigenomic datasets, coverage of histone marks within epigenomic datasets, and the potential for false positives. More datasets from the cell types of interest from added donors would allow for estimations of the variability of chromatin states across the genome. Therefore, allowing researchers to assign confidence to the likelihood of a particular mark in each cell type. Furthermore, the inclusion of epigenomes from RA patients (and not only healthy donors) is likely to identify differential enhancer activity. Related to this limitation is the unknown accuracy of the method for selecting gene and cell type connections for a given GWAS loci. All the proposed connections need to be confirmed with focused experimentation. We demonstrated that H3K4me1 peaks identified more RA GWAS/eQTL overlapping enhancers than H3K27ac. Because of the higher genomic coverage of H3K4me1, it is possible a greater number of these connections are caused by chance.

## Conclusions

To our current knowledge, the study presented here is the most comprehensive dissection of the transcriptional regulation of RA GWAS in immune cell types. We leveraged a large study of DNA variation and gene expression from RA patients to map eQTLs. Then, using this dataset and publicly available epigenome maps, we attempted to deconvolute the RA GWAS loci into potential protein and cell type associations. This integrative analysis provides a bridge to better understand RA GWAS and enable future validation studies to elucidate disease mechanisms.

## Methods

### Patient cohort

RA patient samples were collected from a phase III clinical trial of golimumab in Patients with Active Rheumatoid Arthritis Despite Methotrexate Therapy (GO-FURTHER) [16]. A subset of subjects from the entire trial population at baseline (before golimumab treatment) was utilized (Table 1).

### Peripheral blood gene expression

Peripheral whole blood was collected in PAXgene tubes (Preanalytix, Switzerland). RNA was isolated using the Qiagen Biorobot (Qiagen, Valencia, CA, USA), which followed the protocol from the Qiagen PAXgene MDX kit (cat# 752431), and was modified to collect both total and microRNA. Subject cDNA was amplified through utilization of the NuGEN Ovation Pico WTA System V2 (NuGEN, San Carlos, CA, USA). Microarray hybridization was performed on GeneChip Human Genome U133 Plus 2.0 Array according to the manufacturer’s protocol (Affymetrix, Santa Clara, CA, USA). Data were normalized using Robust Multi-array Average (RMA) algorithm and log base 2 transformed using R. Data are available from NCBI GEO with accession number GSE74143.

Prior to eQTL detection, the gene expression data were adjusted by the first ten principal components (PCs) from principal component analysis and transformed with inverse normal transformation as in previous eQTL studies [14, 18, 53]. The variation captured by these PCs was associated with factors such as sex, age, and technical factors such as RNA quality and microarray batch effect (Additional file 14: Figure S10). We chose to remove ten PCs to balance removing as much unwanted sample differences without also removing genetically determined variation. Removal of 2–200 PCs was tested to choose ten PCs, although the maximal number of *cis*-eQTLs was detected with removal of 50 PCs.

### SNP data from whole-genome sequencing

Genotypes were generated from whole-genome sequencing as described in Standish et al. [17]. DNA isolated from whole blood was sequenced and DNA variants called using a modified GATK pipeline.

### eQTL mapping

eQTL associations were tested for bi-allelic, autosomal SNPs with minor allele frequency (MAF) >0.05, Hardy-Weinberg equilibrium *p* value >10^−6^, and mapping to a reference SNP ID number. The total number of SNPs tested was 6,012,773. Probe sets mapping to non-autosomal chromosomes, not mapping to known genes, or with ambiguous mappings were excluded, leaving 39,515 probe sets for eQTL mapping. After QC of genotype and transcriptomics data, there were 377 matching samples for eQTL mapping. It was confirmed that transcriptomics and DNA samples matched as described previously [54].

Association of SNP genotypes (coded as 0, 1, or 2) and gene expression data was performed using the matrixeQTL R package with a linear model [55]. Subject age and the first three PCs from genotype population stratification (performed with SNPRelate R package) were included as covariates [56]. For *cis*-eQTLs, associations were tested for SNPs less than 1 Mb from a probe set, while *trans*-eQTLs were considered all other distal associations. Multiple hypothesis correction was performed separately for *cis*- and *trans*-eQTLs using permutations as described previously [57].

### Pathway enrichment analysis

Enrichment of gene sets was performed with Nextbio (Illumina, San Diego, CA, USA) with Broad Institute MSigDB canonical pathways for terms in the range of 1–5000 genes in size and the Nextbio Body Atlas for Homo sapiens cell types [58]. All enrichment *p* values were corrected for multiple hypotheses using the Bonferroni correction.

Differentially expressed genes from RA synovial tissue were identified from three publicly available datasets (NCBI GEO dataset IDs: GSE1919 [59], GSE55235 [60], GSE48780 [61]). Conditions were compared using a moderated *t*-test and a *p* value <0.05 cutoff to define gene sets.

### Comparison to GWAS studies

For the comparison to the NHGRI catalog, a snapshot of the catalog was downloaded on 22 December 2014 from the NHGRI website (http://www.genome.gov/gwastudies/). Diseases or traits with ten or more associated SNPs were considered for analysis. *P* values were calculated using a one-sided Fisher’s exact test and adjusted for multiple hypotheses using the Bonferroni correction. The background considered was all SNPs for any disease/trait in the catalog. For the remaining comparisons to RA GWAS results, the 101 RA risk SNPs reported in Okada et al. from the trans-ethnic meta-analysis were used [2]. For each reported SNP, SNPs in LD were defined based on 1000 genomes Project Consortium phase 1 data as those with *r*^2^ ≥ 0.8, using HaploReg (http://www.broadinstitute.org/mammals/haploreg/haploreg_v3.php; [62]).

### Enrichment in tissue-/cell type-specific chromatin states

We compared the locations of the RA eQTLs to regulatory elements of 97 different cell types taken from the Roadmap Epigenomics project and ENCODE [5, 20]. Chromatin states produced by the Roadmap Epigenomics project using six histone modifications (H3K4me3, H3K4me1, H3K27ac, H3K27me3, H3K9me3, and H3K36me3) and ChromHMM [21] were downloaded. The Roadmap Epigenomics project also produced a set of chromatin states using only five marks but including 127 cell types, but we chose not to use this set because H3K27ac was not included and this histone modification has been shown to be the most predictive is enhancer activity [25]. The six histone modifications used produce 18 chromatin states, but many of these states correspond to similar regulatory elements (e.g. “Active TSS” and “Flanking TSS” both represent the TSS); thus to simplify interpretation, the 18 states were collapsed into nine states. The fold-enrichment of the eQTLs in each of the cell type’s chromatin states was calculated by dividing the proportion of total eQTLs that overlapped the state by the proportion of the total genome covered by the state.

To better account for local genomic structure and test whether the enrichment within enhancer regions was statistically significant, we used a recently published algorithm, goshifter [23]. This method uses local permutations of annotation locations (e.g. enhancers marked by H3K27ac) to generate a null distribution and then calculate a *p* value based on the observed overlap of a set of variants (here eQTLs) and those in LD with the given annotation. Enrichment was tested for a given chromatin state annotation for the peak eQTLs (eQTL for each gene with the lowest *p* value). The tests were run using 10^4^ permutations and LD information from the 1000 genomes EUR population with an r^2^ threshold of 0.8 and a window size of 5 × 10^5^ bases.

### Deconvolution of RA GWAS and RA whole blood eQTLs using histone modifications

The deconvolution of RA GWAS hits into cell type and protein associations was carried out for each cell type’s epigenome using a four-step methodology: (1) creating sets of RA GWAS enhancers; (2) creating sets of active genes; (3) using eQTLs to link RA GWAS enhancers and active genes; and (4) adding active genes that overlap the RA GWAS loci.

Creating sets of RA GWAS enhancers was done by downloading lists of genomic loci that are enriched (known as peaks) with H3K27ac and H3K4me1 from the Roadmap Epigenomics project [5]. The Roadmap Epigenomics project had identified these regions by performing chromatin immunoprecipitation followed by high-throughput sequencing (ChIP-seq) using antibodies that are specific to these histone modifications. To facilitate comparison between epigenomes, the Roadmap Project performed all data processing with a common bioinformatics pipeline, which first used Pash 3.0 [63] to map sequencing reads to the genome. Then the program MACS2 [64] was used to identify regions of the genome where significant enrichment of sequence reads group to form peak regions. In this analysis, only peaks with a fold-enrichment >4 were used. From each set we removed any peaks that overlapped (−1500 bp to +500 bp) a transcription start site (TSS; locations obtained from RefSeq [65]) as these likely correspond to promoters and not enhancers (and were handled separately; see below). We used H3K27ac peaks to represent enhancers that are active in a cell type whereas H3K4me1 peaks represent enhancer regions that likely to become active in the cell type upon stimulation or further differentiation. Then to identify the enhancer regions that are associated with RA, we used bedtools [66] to overlap the enhancer sets with the reported RA GWAS SNPs and SNPs that are in LD (*r*^2^ > 0.8). This resulted in 17 to 90 (H3K27ac) and 20 to 138 (H3K4me1) RA GWAS enhancers per cell type.

Creating sets of active genes was done by overlapping their TSSs (−1500 bps to +500 bps) with H3K4me3 (also obtained from the Roadmap Project) or H3K27ac. Only peaks with a fold-enrichment >4 were used. This resulted in between 12,389 and 16,320 active genes per cell type.

Using eQTLs to link RA GWAS enhancers and active genes was done by re-mapping the eQTLs using only RA GWAS enhancers and genes that are active in a particular cell type (as defined above). To do this, we performed eQTL mapping (as described in the “eQTL mapping” section in “Methods” above) using only the SNPs that overlap the RA GWAS enhancers and only the genes that were called as active for each cell type. Thus, RA GWAS enhancers are annotated with one or more genes if there is a significant eQTL association in our dataset and the gene(s) are considered active in the cell type being considered.

Adding active genes that overlap the RA GWAS loci was performed to include GWAS hits that overlap promoters and gene bodies. To do this we used bedtools to identify RA GWAS SNPs and those in LD (*r*^2^ > 0.8) with the gene bodies and promoters of the active genes (−1500 bp from TSS to +500 bp from the transcript termination site) identified in each cell type’s epigenome. This resulted in between 60 and 81 genes in each cell type’s epigenome.

### Network analysis

Network calculations were made as described in Meche et al. using the same publicly available interaction network [28].

### Data visualization

Figures were created using Microsoft Excel, R, Cytoscape, Circos [67], and ArrayStudio (Omicsoft, Cary, NC, USA). Hierarchical clustering as shown in heatmap figures was performed using one minus the Pearson correlation as a distance measure and Ward’s method for linkage. Additional file 10: Figure S6 was created with LocusZoom [68] using “1000 Genomes Nov 2014 EUR” as the LD population.

## Ethics approval

RA patient samples were collected from a phase III clinical trial of golimumab in Patients with Active Rheumatoid Arthritis Despite Methotrexate Therapy (GO-FURTHER). [16] The study (NCT00973479, EudraCT 2008-006064-11) was conducted according to the Declaration of Helsinki and the International Committee on Harmonisation good clinical practices. The protocol was reviewed and approved by each site’s institutional review board or ethics committee. All patients provided written informed consent to genetic and transcriptomics analyses.

## Availability of data and materials

The microarray gene expression data are available from NCBI GEO with accession number GSE74143. The eQTLs are provided as supplemental data files.

## Additional files

The following additional data are available with the online version of this paper: Additional file 1: Contains the *cis*-eQTLs with FDR <5 %. (TXT 104041 kb) Additional file 2: Contains the list of all SNPs tested in the eQTL mapping. (TXT 67993 kb) Additional file 3: Contains the *trans*-eQTLs with FDR <5 %. (TXT 1275 kb) Additional file 4: Figure S1. Comparison of RA *cis*-eQTLs identified in this study to previously published eQTL studies. The eQTLs mapped in this study (“current study, RA blood”) were compared to those from three published studies, labeled by the first author of each study and the cell type or tissues where gene expression was measured. When compared to the current study, each previous study replicated between ~25 % and 50 % of egenes **A** and up to ~30 % of eQTL SNPs **B** reflecting the higher number of SNPs used in this study based on whole-genome sequencing versus the previous studies. When comparing the current study with the previous studies as references, between ~40 % and 60 % of egenes repeated **C** and ~30 % to 50 % of eQTL SNPs replicated **D**. (PDF 7622 kb) Additional file 5: Figure S2. Fold-enrichment of RA eQTLs in chromatin states of diverse tissues. **A** The full results from analysis of 97 datasets. A subset of the full results was shown in Fig. 2c. Both *cis*- and *trans*-eQTLs were compared to positions of the following chromatin states in diverse tissues: active transcription start sites (*TSS*), actively transcribed (*Tx*), enhancers (*Enh*), active enhancers (*EnhA*), heterochromatin (*Het*), bivalent enhancers (*EnhBiv*), bivalent/poised TSS (*TssBiv*), repressed Polycomb (*ReprPC*), and quiescent/low (Quies) regions. PB, peripheral blood. **B** Results from enrichment analysis with peak eQTLs performed using the goshifter method. The values given are the *p* values from permutation and the enrichment in parentheses (number of SNPs overlapping the annotation divided by the total number of SNPs). **C** Enrichment of peak eQTLs in H3K27ac stratified on annotations in other cell types performed using the goshifter method. The *p* values from permutation are shown. (PDF 7890 kb) Additional file 6: Contains the full results of the integration with enhancers described by H3K27ac or H3K4me marks. (XLSX 276 kb) Additional file 7: Figure S3. Genes associated with RA GWAS in T cell specific epigenomic datasets. *Heatmap* of genes associated with RA GWAS SNPs overlapping enhancers in the shown T cell datasets. PB, peripheral blood. Two T cell epigenomes uniquely identify genes that might be explained by unique aspects of the selection markers and (when carried out) in vitro differentiation protocols: (1) “Primary T cells from PB” was the only T cell sample to use CD3+ as a selection marker; and (2) “Primary T helper cells PMA-I stimulated” was the only sample that used Magnetic-activated cell sorting (MACS) [5]. The differences between the two “Primary T helper memory cells from PB” samples might be explained by their different differentiation protocols (number 1 uniquely used CD25M and CD45RO as selection markers) or by the differing donors of origin [5]. (PDF 7665 kb) Additional file 8: Figure S4.
*Heatmap* of genes identified to overlap blood enhancers and RA GWAS loci. *Heatmap* of genes associated with RA GWAS SNPs in primary monocyte, B cell, or T cell datasets. In addition to the associations from overlapping enhancers and eQTLs (as shown in Fig. 3b), genes that overlap RA GWAS loci in the given cell-type datasets are shown. (PDF 7638 kb) Additional file 9: Figure S5.
*PADI4* is active in monocytes but not T cells or B cells. Genome *browser view* of the *PADI4* region with tracks from peripheral blood datasets. Histone modification tracks show the fold-change between ChIP and control read counts. Active histone modifications have *red bars* next to their labels while repressive marks have *blue*. Genes on the positive and negative strands are shown in *blue* and *cyan*, respectively. (PDF 7791 kb) Additional file 10: Figure S6.
*Trans*-eQTLs that overlap RA risk loci on chromosome 6. Genome browser view showing eQTL peaks for three genes associated distally with RA GWAS loci in the HLA region. For each gene, the most significant eQTLs are shown. The color of the eQTL markers corresponds to LD r^2^ in reference to the strongest eQTL for each gene (as labeled and data shown to the *right*). The *yellow* highlights the position of the strongest eQTL for each gene. Histone modification tracks show the fold-change between ChIP and control read counts. Active histone modifications have *red bars* next to their labels while repressive marks have *blue*. (PDF 7709 kb) Additional file 11: Figure S7. Enrichment of RA-associated genes including genes for those RA GWAS SNPs that were not annotated with our approach. Genes identified as associated with RA GWAS SNPs in monocytes, B cells, or T cells were tested for enrichment with the MSigDB term database. This corresponds to Fig. 4a, except including additional genes for the RA GWAS SNPs that were not identified with our approach using the monocyte, B cell, and T cell datasets. Genes were chosen for each SNP based on previously published annotation [2]. (PDF 7609 kb) Additional file 12: Figure S8. Network connectivity and neighborhoods of genes identified through overlap with enhancers and active genes in primary B cell, T cell, and monocyte datasets. **A** Network connectivity parameters compared for GWAS-associated genes identified in this study compared to those annotated by NCBI phenotype-genotype integrator. **B** Enrichment of interactome subnetwork genes in cell types and canonical pathways. **C** Subnetwork of genes that were found to be unique to T cells. **D** Enrichment of T cell specific interactome subnetwork genes in cell types and canonical pathways [2, 28]. (ZIP 7.33 mb) Additional file 13: Figure S9. Comparison of RA eQTLs to healthy donor eQTLs. **A** Comparison of allele frequencies in the RA population used in this study compared to the 1000 genomes dataset for those SNPs that were eQTLs in our dataset. The allele frequency of the peak eQTL for every egene (SNP with lowest *p* value) was compared to the 1000 genomes project dataset (accessed with Haploreg; http://www.broadinstitute.org/mammals/haploreg/haploreg_v3.php). **B** Comparison of allele frequencies for all peak eQTLs in our RA population versus the 1000 genomes EUR population. **C** Comparison of allele frequencies for peak eQTLs that overlap RA GWAS loci or those in high LD (GWAS loci from Okada et al. [2]). **D** Overlap of egenes in our RA population and the GTEx whole blood dataset (v6). **E** Comparison of enrichment in RA synovium datasets for egenes that were unique to the RA dataset, the GTEx dataset, or common to both. These datasets used were: RA synovium compared to healthy controls (GSE1919), inflamed RA synovium compared to non-inflamed controls (GSE48780), and RA synovium compared to healthy controls (GSE55235). The *p* values were generated by a one-sided Fisher’s exact test as described in the manuscript methods. **F** Selection of enrichment results for the egenes using Nextbio body atlas. The top results for each gene list are shown. The *p* values are adjusted using the Bonferroni correction. (PDF 7730 kb) Additional file 14: Figure S10. PCs removed from gene expression data are correlated with technical and non-biological sources of variation. The spearman correlation coefficient between sample metadata and gene expression PCs is displayed for the first 20 PCs derived from the whole blood gene expression data. (PDF 7591 kb)

## References

[1] McInnes IB, Schett G (2011). The pathogenesis of rheumatoid arthritis. N Engl J Med..

[2] Okada Y, Wu D, Trynka G, Raj T, Terao C, Ikari K (2014). Genetics of rheumatoid arthritis contributes to biology and drug discovery. Nature..

[3] Maurano MT, Humbert R, Rynes E, Thurman RE, Haugen E, Wang H (2012). Systematic localization of common disease-associated variation in regulatory DNA. Science..

[4] Farh KK, Marson A, Zhu J, Kleinewietfeld M, Housley WJ, Beik S (2015). Genetic and epigenetic fine mapping of causal autoimmune disease variants. Nature..

[5] Kundaje A, Meuleman W, Ernst J, Bilenky M, Yen A, Roadmap Epigenomics Consortium (2015). Integrative analysis of 111 reference human epigenomes. Nature.

[6] Freudenberg J, Gregersen P, Li W (2015). Enrichment of genetic variants for rheumatoid arthritis within T-cell and NK-cell enhancer regions. Mol Med..

[7] Trynka G, Sandor C, Han B, Xu H, Stranger BE, Liu XS (2013). Chromatin marks identify critical cell types for fine mapping complex trait variants. Nat Genet..

[8] Musunuru K, Strong A, Frank-Kamenetsky M, Lee NE, Ahfeldt T, Sachs KV (2010). From noncoding variant to phenotype via SORT1 at the 1p13 cholesterol locus. Nature..

[9] Harismendy O, Notani D, Song X, Rahim NG, Tanasa B, Heintzman N (2011). 9p21 DNA variants associated with coronary artery disease impair interferon-gamma signalling response. Nature..

[10] Nicolae DL, Gamazon E, Zhang W, Duan S, Dolan ME, Cox NJ (2010). Trait-associated SNPs are more likely to be eQTLs: annotation to enhance discovery from GWAS. PLoS Genet..

[11] Lamontagne M, Timens W, Hao K, Bosse Y, Laviolette M, Steiling K (2014). Genetic regulation of gene expression in the lung identifies CST3 and CD22 as potential causal genes for airflow obstruction. Thorax..

[12] Whitaker JW, Nguyen TT, Zhu Y, Wildberg A, Wang W (2015). Computational schemes for the prediction and annotation of enhancers from epigenomic assays. Methods..

[13] Fairfax BP, Makino S, Radhakrishnan J, Plant K, Leslie S, Dilthey A (2012). Genetics of gene expression in primary immune cells identifies cell type-specific master regulators and roles of HLA alleles. Nat Genet..

[14] Raj T, Rothamel K, Mostafavi S, Ye C, Lee MN, Replogle JM (2014). Polarization of the effects of autoimmune and neurodegenerative risk alleles in leukocytes. Science..

[15] GTEx Consortium (2015). Human genomics. The Genotype-Tissue Expression (GTEx) pilot analysis: multitissue gene regulation in humans. Science.

[16] Weinblatt ME, Bingham CO, Mendelsohn AM, Kim L, Mack M, Lu J (2013). Intravenous golimumab is effective in patients with active rheumatoid arthritis despite methotrexate therapy with responses as early as week 2: results of the phase 3, randomised, multicentre, double-blind, placebo-controlled GO-FURTHER trial. Ann Rheum Dis.

[17] Standish KA, Carland TM, Lockwood GK, Pfeiffer W, Tatineni M, Huang CC (2015). Group-based variant calling leveraging next-generation supercomputing for large-scale whole-genome sequencing studies. BMC Bioinformatics..

[18] Fehrmann RS, Jansen RC, Veldink JH, Westra HJ, Arends D, Bonder MJ (2011). Trans-eQTLs reveal that independent genetic variants associated with a complex phenotype converge on intermediate genes, with a major role for the HLA. PLoS Genet..

[19] Welter D, MacArthur J, Morales J, Burdett T, Hall P, Junkins H (2014). The NHGRI GWAS Catalog, a curated resource of SNP-trait associations. Nucleic Acids Res..

[20] Encode Project Consortium (2012). An integrated encyclopedia of DNA elements in the human genome. Nature.

[21] Ernst J, Kellis M (2010). Discovery and characterization of chromatin states for systematic annotation of the human genome. Nat Biotechnol..

[22] Heintzman ND, Hon GC, Hawkins RD, Kheradpour P, Stark A, Harp LF (2009). Histone modifications at human enhancers reflect global cell-type-specific gene expression. Nature..

[23] Trynka G, Westra HJ, Slowikowski K, Hu X, Xu H, Stranger BE (2015). Disentangling the effects of colocalizing genomic annotations to functionally prioritize non-coding variants within complex-trait loci. Am J Hum Genet..

[24] Heinz S, Romanoski CE, Benner C, Glass CK (2015). The selection and function of cell type-specific enhancers. Nat Rev Mol Cell Biol..

[25] Creyghton MP, Cheng AW, Welstead GG, Kooistra T, Carey BW, Steine EJ (2010). Histone H3K27ac separates active from poised enhancers and predicts developmental state. Proc Natl Acad Sci U S A..

[26] Ostuni R, Piccolo V, Barozzi I, Polletti S, Termanini A, Bonifacio S (2013). Latent enhancers activated by stimulation in differentiated cells. Cell..

[27] Sakabe NJ, Savic D, Nobrega MA (2012). Transcriptional enhancers in development and disease. Genome Biol..

[28] Menche J, Sharma A, Kitsak M, Ghiassian SD, Vidal M, Loscalzo J (2015). Disease networks. Uncovering disease-disease relationships through the incomplete interactome. Science.

[29] Greene CS, Krishnan A, Wong AK, Ricciotti E, Zelaya RA, Himmelstein DS (2015). Understanding multicellular function and disease with human tissue-specific networks. Nat Genet..

[30] Califano A, Butte AJ, Friend S, Ideker T, Schadt E (2012). Leveraging models of cell regulation and GWAS data in integrative network-based association studies. Nat Genet..

[31] Poelmans G, Pauls DL, Buitelaar JK, Franke B (2011). Integrated genome-wide association study findings: identification of a neurodevelopmental network for attention deficit hyperactivity disorder. Am J Psychiatry..

[32] Shen H, Goodall JC, Hill Gaston JS (2009). Frequency and phenotype of peripheral blood Th17 cells in ankylosing spondylitis and rheumatoid arthritis. Arthritis Rheum..

[33] van Amelsfort JM, Jacobs KM, Bijlsma JW, Lafeber FP, Taams LS (2004). CD4(+)CD25(+) regulatory T cells in rheumatoid arthritis: differences in the presence, phenotype, and function between peripheral blood and synovial fluid. Arthritis Rheum..

[34] Ptacek J, Hawtin RE, Louie B, Evensen E, Cordeiro J, Mittleman B (2013). Novel biomarkers from peripheral blood mononuclear cells indicate disease activity in rheumatoid arthritis patients. Arthritis Rheum.

[35] Fairfax BP, Humburg P, Makino S, Naranbhai V, Wong D, Lau E (2014). Innate immune activity conditions the effect of regulatory variants upon monocyte gene expression. Science..

[36] Begovich AB, Carlton VE, Honigberg LA, Schrodi SJ, Chokkalingam AP, Alexander HC (2004). A missense single-nucleotide polymorphism in a gene encoding a protein tyrosine phosphatase (PTPN22) is associated with rheumatoid arthritis. Am J Hum Genet..

[37] Hashimoto Y, Kakegawa H, Narita Y, Hachiya Y, Hayakawa T, Kos J (2001). Significance of cathepsin B accumulation in synovial fluid of rheumatoid arthritis. Biochem Biophys Res Commun..

[38] Davis RS (2007). Fc receptor-like molecules. Annu Rev Immunol..

[39] Haga CL, Ehrhardt GR, Boohaker RJ, Davis RS, Cooper MD (2007). Fc receptor-like 5 inhibits B cell activation via SHP-1 tyrosine phosphatase recruitment. Proc Natl Acad Sci U S A..

[40] Franco A, Damdinsuren B, Ise T, Dement-Brown J, Li H, Nagata S (2013). Human Fc receptor-like 5 binds intact IgG via mechanisms distinct from those of Fc receptors. J Immunol..

[41] Owczarczyk K, Lal P, Abbas AR, Wolslegel K, Holweg CT, Dummer W (2011). A plasmablast biomarker for nonresponse to antibody therapy to CD20 in rheumatoid arthritis. Sci Transl Med.

[42] Catalan D, Aravena O, Sabugo F, Wurmann P, Soto L, Kalergis AM (2010). B cells from rheumatoid arthritis patients show important alterations in the expression of CD86 and FcgammaRIIb, which are modulated by anti-tumor necrosis factor therapy. Arthritis Res Ther..

[43] Ranzani V, Rossetti G, Panzeri I, Arrigoni A, Bonnal RJ, Curti S (2015). The long intergenic noncoding RNA landscape of human lymphocytes highlights the regulation of T cell differentiation by linc-MAF-4. Nat Immunol..

[44] Eyre S, Hinks A, Bowes J, Flynn E, Martin P, Wilson AG (2010). Overlapping genetic susceptibility variants between three autoimmune disorders: rheumatoid arthritis, type 1 diabetes and coeliac disease. Arthritis Res Ther..

[45] Bartok B, Firestein GS (2010). Fibroblast-like synoviocytes: key effector cells in rheumatoid arthritis. Immunol Rev..

[46] Nakano K, Whitaker JW, Boyle DL, Wang W, Firestein GS (2013). DNA methylome signature in rheumatoid arthritis. Ann Rheum Dis..

[47] Whitaker JW, Shoemaker R, Boyle DL, Hillman J, Anderson D, Wang W (2013). An imprinted rheumatoid arthritis methylome signature reflects pathogenic phenotype. Genome Med..

[48] Ai R, Whitaker JW, Boyle DL, Tak PP, Gerlag DM, Wang W (2015). DNA methylome signature in early rheumatoid arthritis synoviocytes compared with longstanding rheumatoid arthritis synoviocytes. Arthritis Rheumatol..

[49] Whitaker JW, Boyle DL, Bartok B, Ball ST, Gay S, Wang W (2015). Integrative omics analysis of rheumatoid arthritis identifies non-obvious therapeutic targets. PLoS One..

[50] Ekwall AK, Whitaker JW, Hammaker D, Bugbee WD, Wang W, Firestein GS (2015). The rheumatoid arthritis risk gene LBH regulates growth in fibroblast-like synoviocytes. Arthritis Rheumatol..

[51] Kelly S, Humby F, Filer A, Ng N, Di Cicco M, Hands RE (2015). Ultrasound-guided synovial biopsy: a safe, well-tolerated and reliable technique for obtaining high-quality synovial tissue from both large and small joints in early arthritis patients. Ann Rheum Dis..

[52] Giambartolomei C, Vukcevic D, Schadt EE, Franke L, Hingorani AD, Wallace C (2014). Bayesian test for colocalisation between pairs of genetic association studies using summary statistics. PLoS Genet..

[53] Stegle O, Parts L, Durbin R, Winn J (2010). A Bayesian framework to account for complex non-genetic factors in gene expression levels greatly increases power in eQTL studies. PLoS Comput Biol..

[54] Schadt EE, Woo S, Hao K (2012). Bayesian method to predict individual SNP genotypes from gene expression data. Nat Genet..

[55] Shabalin AA (2012). Matrix eQTL: ultra fast eQTL analysis via large matrix operations. Bioinformatics..

[56] Zheng X, Levine D, Shen J, Gogarten SM, Laurie C, Weir BS (2012). A high-performance computing toolset for relatedness and principal component analysis of SNP data. Bioinformatics..

[57] Schadt EE, Molony C, Chudin E, Hao K, Yang X, Lum PY (2008). Mapping the genetic architecture of gene expression in human liver. PLoS Biol..

[58] Kupershmidt I, Su QJ, Grewal A, Sundaresh S, Halperin I, Flynn J (2010). Ontology-based meta-analysis of global collections of high-throughput public data. PLoS One..

[59] Ungethuem U, Haeupl T, Witt H, Koczan D, Krenn V, Huber H (2010). Molecular signatures and new candidates to target the pathogenesis of rheumatoid arthritis. Physiol Genomics..

[60] Woetzel D, Huber R, Kupfer P, Pohlers D, Pfaff M, Driesch D (2014). Identification of rheumatoid arthritis and osteoarthritis patients by transcriptome-based rule set generation. Arthritis Res Ther..

[61] Sun Y, Caplazi P, Zhang J, Mazloom A, Kummerfeld S, Quinones G (2014). PILRalpha negatively regulates mouse inflammatory arthritis. J Immunol..

[62] Ward LD, Kellis M (2012). HaploReg: a resource for exploring chromatin states, conservation, and regulatory motif alterations within sets of genetically linked variants. Nucleic Acids Res..

[63] Coarfa C, Yu F, Miller CA, Chen Z, Harris RA, Milosavljevic A (2010). Pash 3.0: A versatile software package for read mapping and integrative analysis of genomic and epigenomic variation using massively parallel DNA sequencing. BMC Bioinformatics..

[64] Zhang Y, Liu T, Meyer CA, Eeckhoute J, Johnson DS, Bernstein BE (2008). Model-based analysis of ChIP-Seq (MACS). Genome Biol..

[65] Pruitt KD, Brown GR, Hiatt SM, Thibaud-Nissen F, Astashyn A, Ermolaeva O (2014). RefSeq: an update on mammalian reference sequences. Nucleic Acids Res..

[66] Quinlan AR, Hall IM (2010). BEDTools: a flexible suite of utilities for comparing genomic features. Bioinformatics..

[67] Krzywinski M, Schein J, Birol I, Connors J, Gascoyne R, Horsman D (2009). Circos: an information aesthetic for comparative genomics. Genome Res..

[68] Pruim RJ, Welch RP, Sanna S, Teslovich TM, Chines PS, Gliedt TP (2010). LocusZoom: regional visualization of genome-wide association scan results. Bioinformatics..

