# Supplementary figures and images for: Integrative genomic deconvolution of rheumatoid arthritis GWAS loci into gene and cell type associations
Source: Genome Biol. 2016 Apr 30;17:79. doi: 10.1186/s13059-016-0948-6 (PMC4853861; doi:10.1186/s13059-016-0948-6)

Figure S1:  
comparison of other eQTL studies to our study

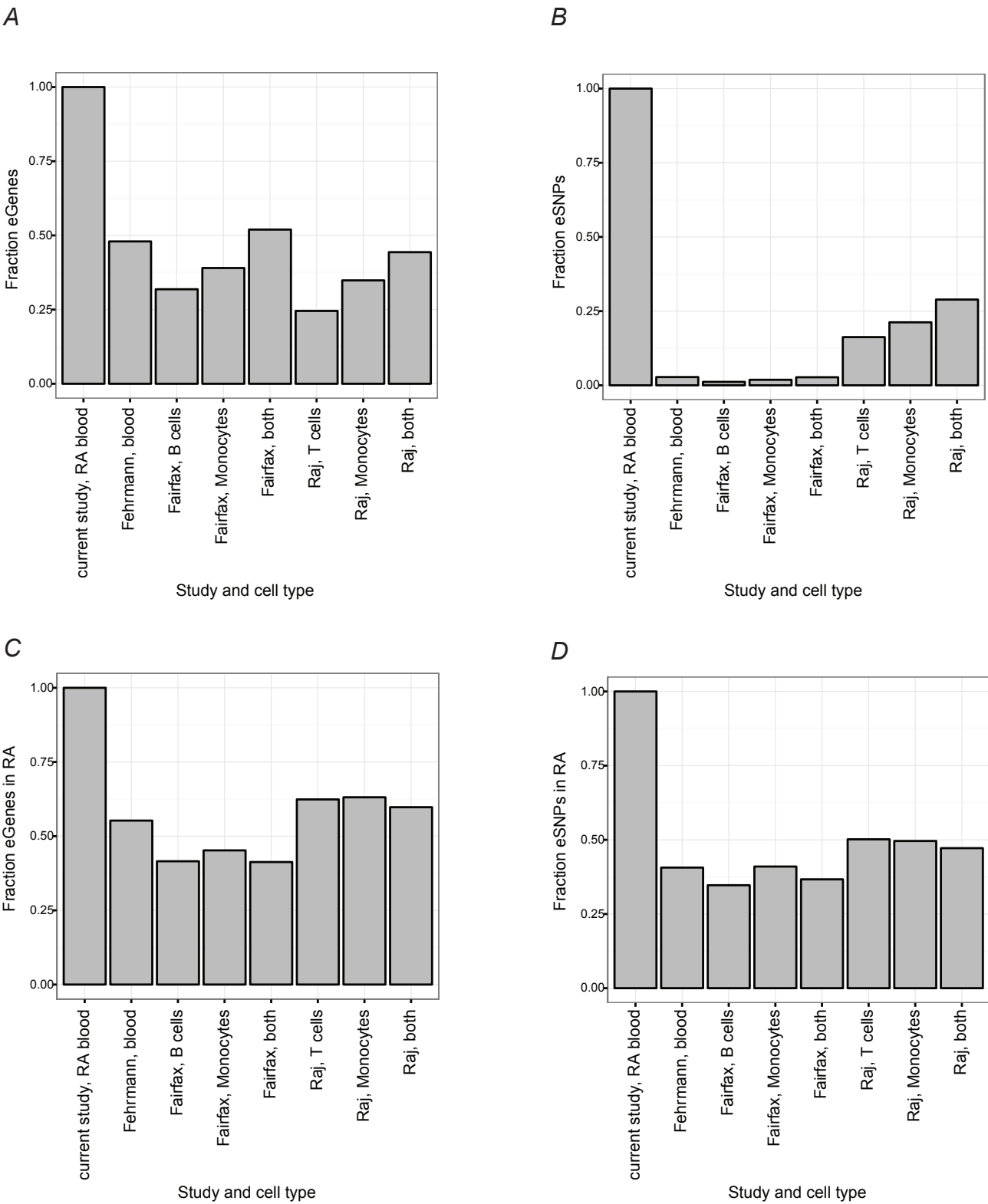

Supplement: Additional file 4: Figure S1. — Comparison of RA cis-eQTLs identified in this study to previously published eQTL studies. The eQTLs mapped in this study (“current study, RA blood”) were compared to those from three published studies, labeled by the first author of each study and the cell type or tissues where gene expression was measured. When compared to the current study, each previous study replicated between ~25 % and 50 % of egenes A and up to ~30 % of eQTL SNPs B reflecting the higher number of SNPs used in this study based on whole-genome sequencing versus the previous studies. When comparing the current study with the previous studies as references, between ~40 % and 60 % of egenes repeated C and ~30 % to 50 % of eQTL SNPs replicated D. (PDF 7622 kb) [file 13059_2016_948_MOESM4_ESM.pdf]

Figure S3

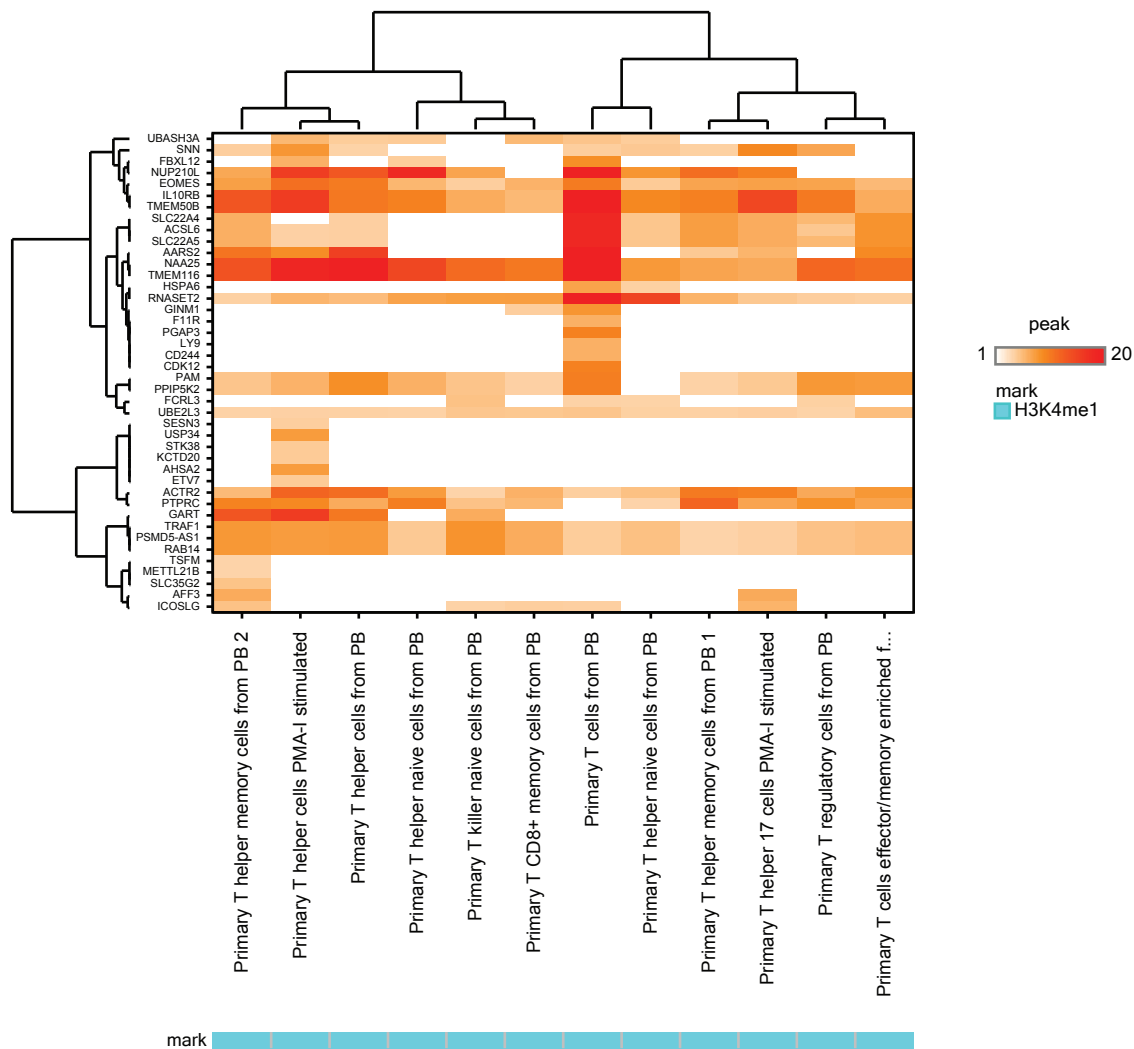

Supplement: Additional file 7: Figure S3. — Genes associated with RA GWAS in T cell specific epigenomic datasets. Heatmap of genes associated with RA GWAS SNPs overlapping enhancers in the shown T cell datasets. PB, peripheral blood. Two T cell epigenomes uniquely identify genes that might be explained by unique aspects of the selection markers and (when carried out) in vitro differentiation protocols: (1) “Primary T cells from PB” was the only T cell sample to use CD3+ as a selection marker; and (2) “Primary T helper cells PMA-I stimulated” was the only sample that used Magnetic-activated cell sorting (MACS) [5]. The differences between the two “Primary T helper memory cells from PB” samples might be explained by their different differentiation protocols (number 1 uniquely used CD25M and CD45RO as selection markers) or by the differing donors of origin [5]. (PDF 7665 kb) [file 13059_2016_948_MOESM7_ESM.pdf]

Figure S4

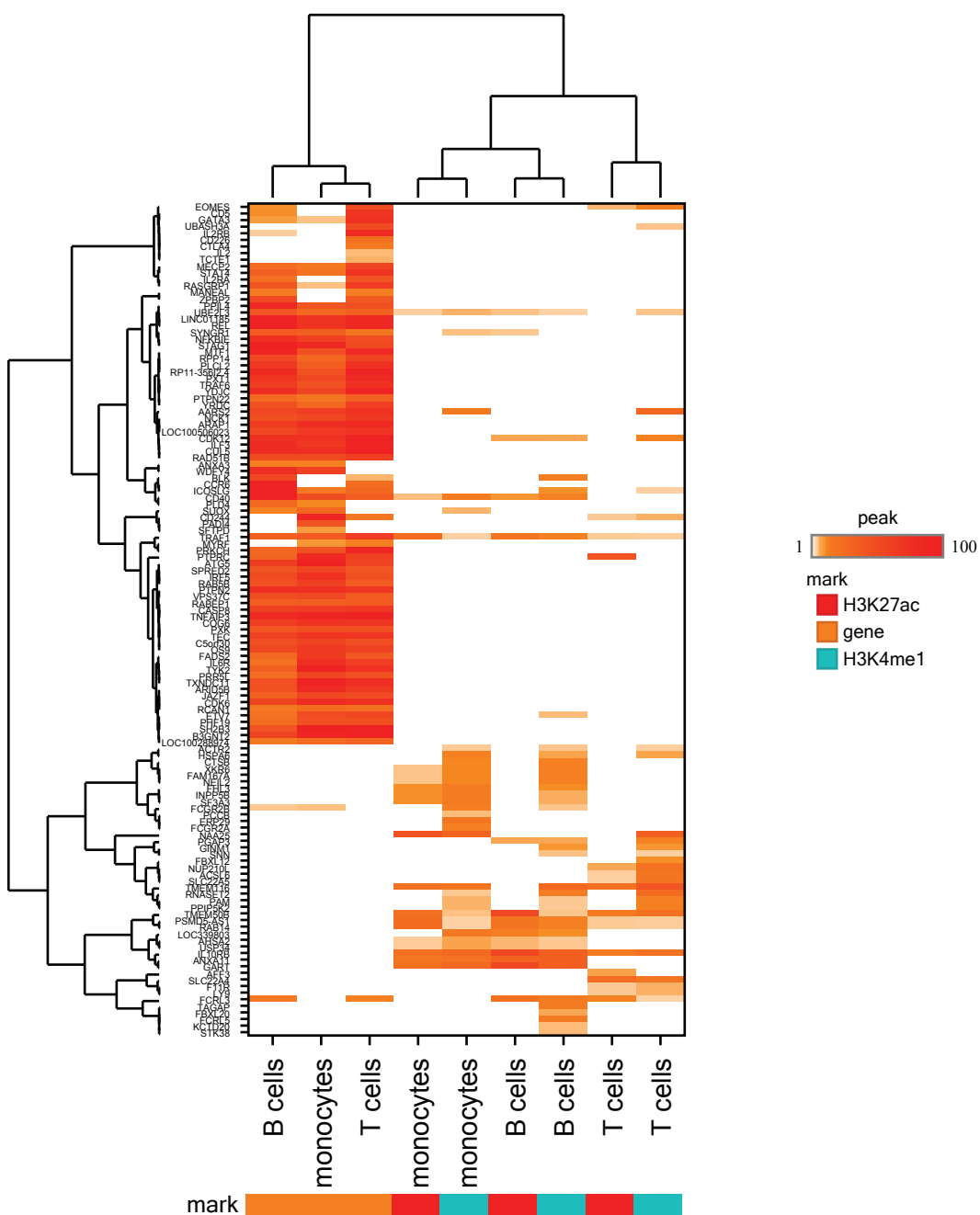

Supplement: Additional file 8: Figure S4. — Heatmap of genes identified to overlap blood enhancers and RA GWAS loci. Heatmap of genes associated with RA GWAS SNPs in primary monocyte, B cell, or T cell datasets. In addition to the associations from overlapping enhancers and eQTLs (as shown in Fig. 3b), genes that overlap RA GWAS loci in the given cell-type datasets are shown. (PDF 7638 kb) [file 13059_2016_948_MOESM8_ESM.pdf]

Figure S5

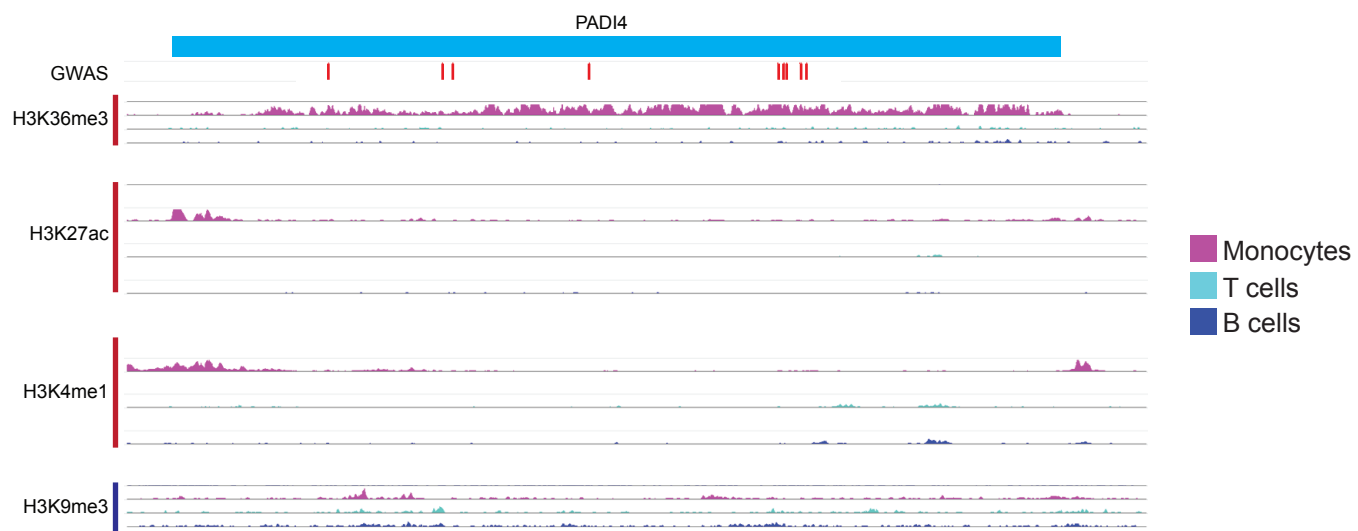

Supplement: Additional file 9: Figure S5. — PADI4 is active in monocytes but not T cells or B cells. Genome browser view of the PADI4 region with tracks from peripheral blood datasets. Histone modification tracks show the fold-change between ChIP and control read counts. Active histone modifications have red bars next to their labels while repressive marks have blue. Genes on the positive and negative strands are shown in blue and cyan, respectively. (PDF 7791 kb) [file 13059_2016_948_MOESM9_ESM.pdf]

Figure S6

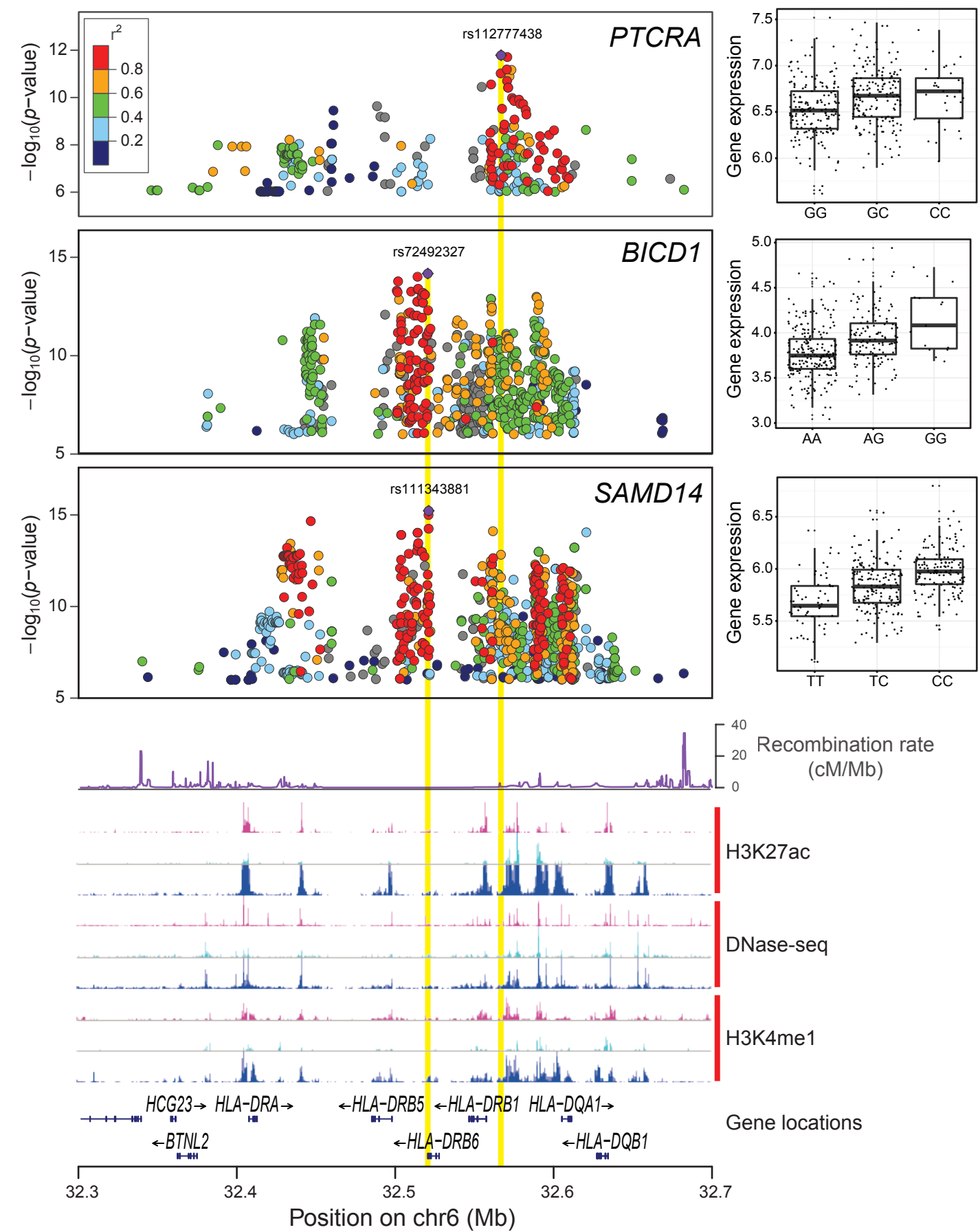

Supplement: Additional file 10: Figure S6. — Trans-eQTLs that overlap RA risk loci on chromosome 6. Genome browser view showing eQTL peaks for three genes associated distally with RA GWAS loci in the HLA region. For each gene, the most significant eQTLs are shown. The color of the eQTL markers corresponds to LD r2 in reference to the strongest eQTL for each gene (as labeled and data shown to the right). The yellow highlights the position of the strongest eQTL for each gene. Histone modification tracks show the fold-change between ChIP and control read counts. Active histone modifications have red bars next to their labels while repressive marks have blue. (PDF 7709 kb) [file 13059_2016_948_MOESM10_ESM.pdf]

Figure S7

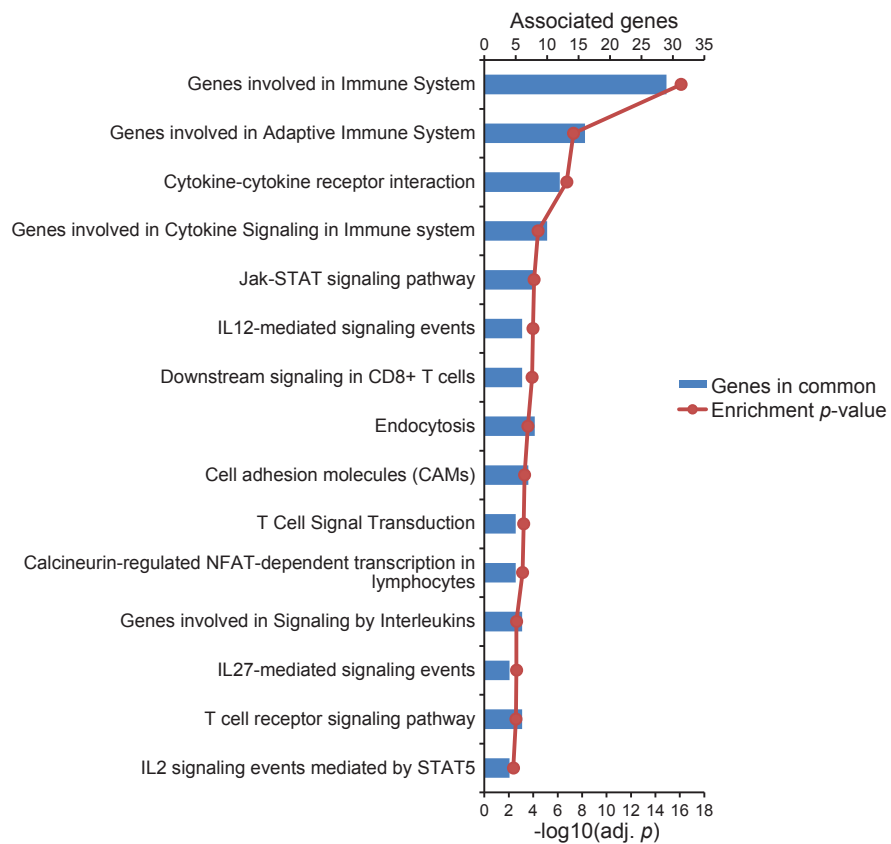

Supplement: Additional file 11: Figure S7. — Enrichment of RA-associated genes including genes for those RA GWAS SNPs that were not annotated with our approach. Genes identified as associated with RA GWAS SNPs in monocytes, B cells, or T cells were tested for enrichment with the MSigDB term database. This corresponds to Fig. 4a, except including additional genes for the RA GWAS SNPs that were not identified with our approach using the monocyte, B cell, and T cell datasets. Genes were chosen for each SNP based on previously published annotation [2]. (PDF 7609 kb) [file 13059_2016_948_MOESM11_ESM.pdf]

Figure S8

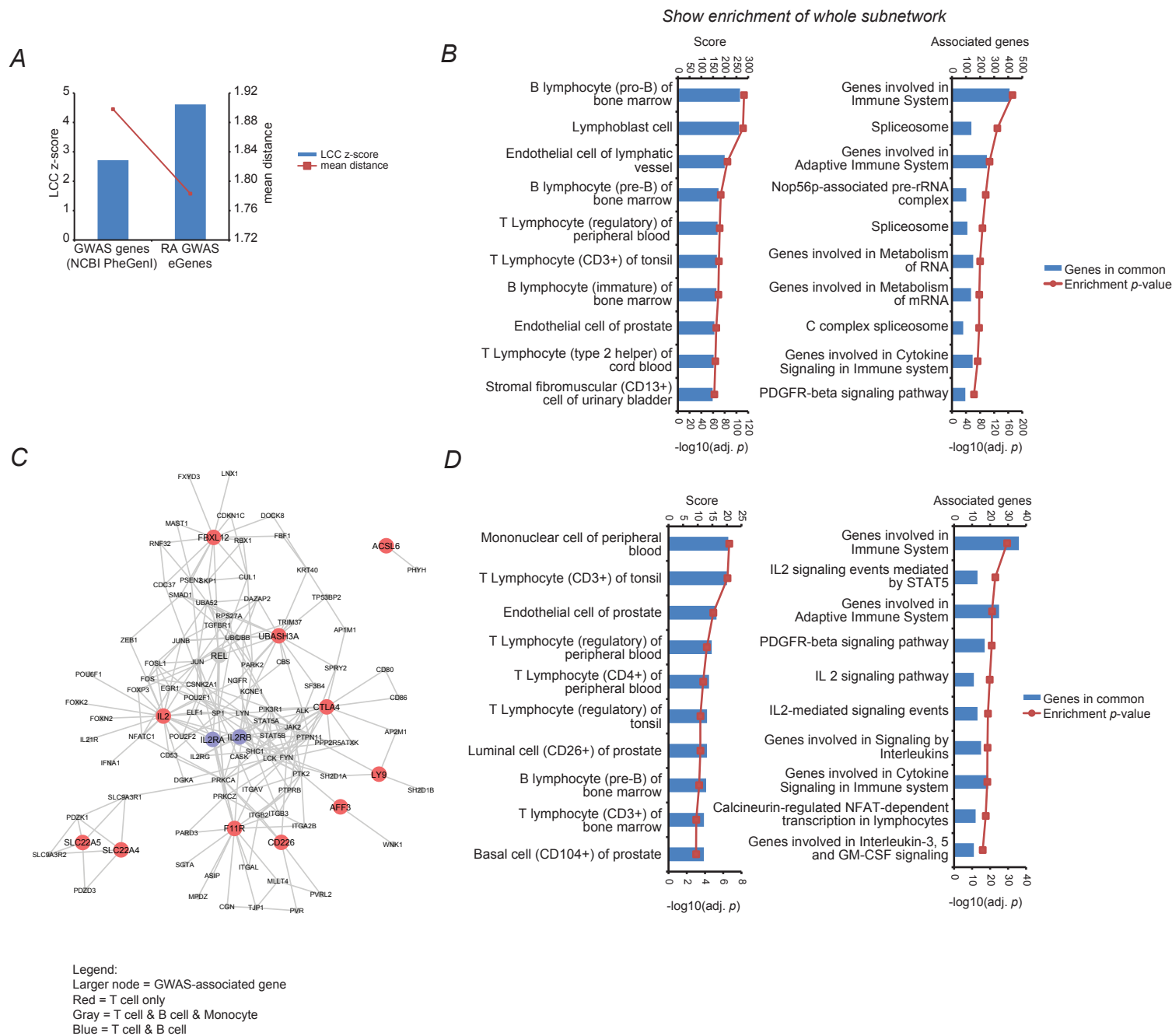

Supplement: Additional file 12: Figure S8. — Network connectivity and neighborhoods of genes identified through overlap with enhancers and active genes in primary B cell, T cell, and monocyte datasets. A Network connectivity parameters compared for GWAS-associated genes identified in this study compared to those annotated by NCBI phenotype-genotype integrator. B Enrichment of interactome subnetwork genes in cell types and canonical pathways. C Subnetwork of genes that were found to be unique to T cells. D Enrichment of T cell specific interactome subnetwork genes in cell types and canonical pathways [2, 28]. (ZIP 7.33 mb) [file 13059_2016_948_MOESM12_ESM.zip › 13059_2016_948_MOESM12_ESM/13059_2016_948_MOESM12_ESM.pdf]

Figure S10

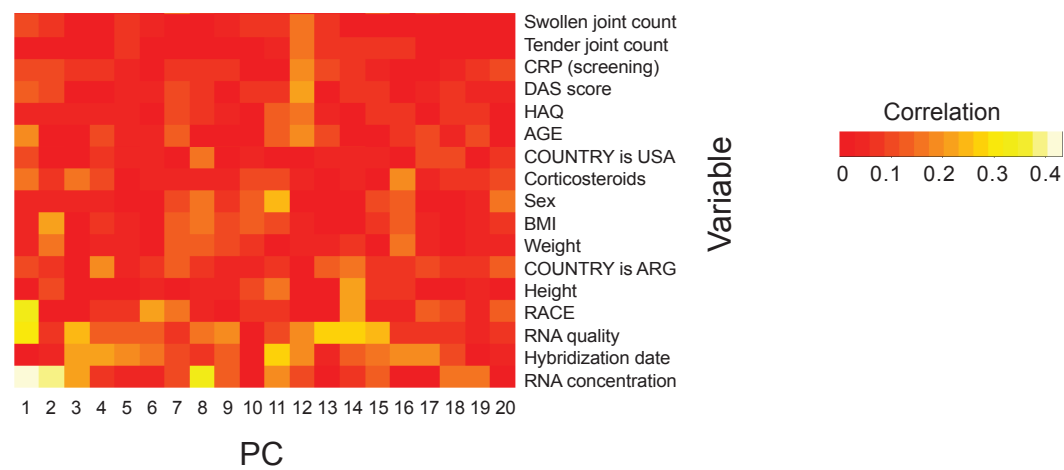

Supplement: Additional file 14: Figure S10. — PCs removed from gene expression data are correlated with technical and non-biological sources of variation. The spearman correlation coefficient between sample metadata and gene expression PCs is displayed for the first 20 PCs derived from the whole blood gene expression data. (PDF 7591 kb) [file 13059_2016_948_MOESM14_ESM.pdf]
