# Supplementary material for: Integrative genomic deconvolution of rheumatoid arthritis GWAS loci into gene and cell type associations
Source: Genome Biol. 2016 Apr 30;17:79. doi: 10.1186/s13059-016-0948-6 (PMC4853861; doi:10.1186/s13059-016-0948-6)
Supplement: Additional file 5: Figure S2. — Fold-enrichment of RA eQTLs in chromatin states of diverse tissues. A The full results from analysis of 97 datasets. A subset of the full results was shown in Fig. 2c. Both cis- and trans-eQTLs were compared to positions of the following chromatin states in diverse tissues: active transcription start sites (TSS), actively transcribed (Tx), enhancers (Enh), active enhancers (EnhA), heterochromatin (Het), bivalent enhancers (EnhBiv), bivalent/poised TSS (TssBiv), repressed Polycomb (ReprPC), and quiescent/low (Quies) regions. PB, peripheral blood. B Results from enrichment analysis with peak eQTLs performed using the goshifter method. The values given are the p values from permutation and the enrichment in parentheses (number of SNPs overlapping the annotation divided by the total number of SNPs). C Enrichment of peak eQTLs in H3K27ac stratified on annotations in other cell types performed using the goshifter method. The p values from permutation are shown. (PDF 7890 kb) [file 13059_2016_948_MOESM5_ESM.pdf]

Figure S2

A

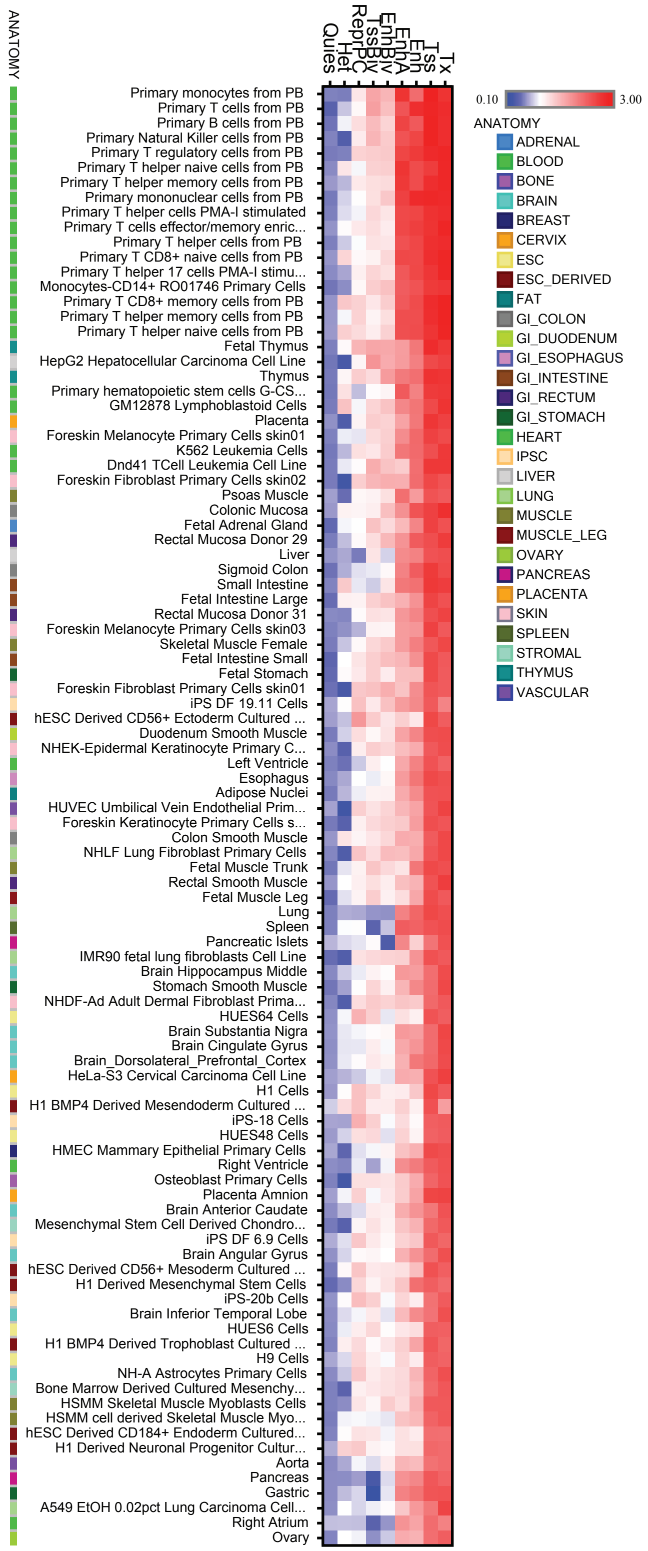

B

Cell/tissue	*p*-value H3K4me1	*p*-value H3K27ac
T cell	0.0034 (0.5084)	<1e-4 (0.3732)
B cell	0.0716 (0.5027)	<1e-4 (0.3439)
monocyte	0.0045 (0.5229)	<1e-4 (0.3893)
ovary	0.8745 (0.1619)	0.0074 (0.1875)
right atrium	0.7114 (0.2122)	<1e-4 (0.3394)
A549	0.6037 (0.3132)	<1e-4 (0.3791)

C

Cell/tissue	*p*-value H3K27ac conditioned on nothing	*p*-value H3K27ac conditioned on T cell	*p*-value H3K27ac conditioned on B cell	*p*-value H3K27ac conditioned on monocyte
T cell	<1e-4	-	0.4943	0.2411
B cell	<1e-4	0.0047	-	0.0748
monocyte	<1e-4	<1e-4	<1e-4	-
ovary	0.0074	0.179	0.243	0.378
right atrium	<1e-4	0.0147	0.129	0.3063
A549	<1e-4	0.0275	0.0852	0.1674
