# Supplementary material for: Integrative genomic deconvolution of rheumatoid arthritis GWAS loci into gene and cell type associations
Source: Genome Biol. 2016 Apr 30;17:79. doi: 10.1186/s13059-016-0948-6 (PMC4853861; doi:10.1186/s13059-016-0948-6)
Supplement: Additional file 12: Figure S8. — Network connectivity and neighborhoods of genes identified through overlap with enhancers and active genes in primary B cell, T cell, and monocyte datasets. A Network connectivity parameters compared for GWAS-associated genes identified in this study compared to those annotated by NCBI phenotype-genotype integrator. B Enrichment of interactome subnetwork genes in cell types and canonical pathways. C Subnetwork of genes that were found to be unique to T cells. D Enrichment of T cell specific interactome subnetwork genes in cell types and canonical pathways [2, 28]. (ZIP 7.33 mb) [file 13059_2016_948_MOESM12_ESM.zip › 13059_2016_948_MOESM12_ESM/13059_2016_948_MOESM12_ESM.docx]

Additional file 12: Figure S8

Introduction:

This network-based approach supposes that highly connected genes are functionally related. We hypothesized that the RA-related genes we identified by integrating eQTLs from RA patients and epigenomic mapping would form a more highly connected component on a gene interaction network than GWAS-associated genes annotated from GWAS SNPs with a traditional approach. For this comparison, a publicly available human interactome was utilized [[1](#_ENREF_1)]. We calculated the largest connected component formed by RA disease genes on this network. These are genes that are directly linked to one another. We also calculated the mean shortest distance between disease genes on the network. This is a metric of the diameter of the disease genes on the network and demonstrates how directly connected on average each pairwise combination of disease genes are.

To examine the relevant biology of the neighborhood the RA GWAS-associated genes occupy on the interactome, we created a subnetwork of genes that included the GWAS-associated genes and all their first neighbors, resulting in a subnetwork of 2384 genes. This subnetwork contains many genes known to be important for RA disease etiology, including several validated drug targets, including *TNF*, *MS4A1* (CD20), *IL6R*, and *JAK2*. In total, there are 14 genes whose gene products are known to be targets of drugs used clinically for the treatment of RA [[2](#_ENREF_2)]. The subnetwork genes were enriched for genes that are highly expressed in B cell and T cell subtypes as well as pathways such as “Genes involved in Adaptive Immune System” and “Genes involved in Cytokine Signaling in immune system” (Additional file 13: Figure S9B).

We examined the network neighborhood of the GWAS-associated genes identified to be potentially T cell specific (unique to T cell dataset and not B cell or monocyte dataset) (Additional file 13: Figure S9C). Key regulator analysis identified *IL2*, *F11R*, and *UBASH3A* as the top ranked nodes in this subnetwork [[3](#_ENREF_3)]. As was performed for the entire RA subnetwork, the member genes were evaluated for enrichment in cell types and canonical pathways (Additional file 13: Figure S9D). The pathways identified focus on the immune system and IL2 signaling in particular.

References:

1. Menche J, Sharma A, Kitsak M, Ghiassian SD, Vidal M, Loscalzo J, Barabasi AL: **Disease networks. Uncovering disease-disease relationships through the incomplete interactome.** *Science* 2015, **347:**1257601.

2. Okada Y, Wu D, Trynka G, Raj T, Terao C, Ikari K, Kochi Y, Ohmura K, Suzuki A, Yoshida S, et al: **Genetics of rheumatoid arthritis contributes to biology and drug discovery.** *Nature* 2014, **506:**376-381.

3. Zhu J, Zhang B, Smith EN, Drees B, Brem RB, Kruglyak L, Bumgarner RE, Schadt EE: **Integrating large-scale functional genomic data to dissect the complexity of yeast regulatory networks.** *Nat Genet* 2008, **40:**854-861.
