# Supplementary material for: Integrative genomic deconvolution of rheumatoid arthritis GWAS loci into gene and cell type associations
Source: Genome Biol. 2016 Apr 30;17:79. doi: 10.1186/s13059-016-0948-6 (PMC4853861; doi:10.1186/s13059-016-0948-6)
Supplement: Additional file 13: Figure S9. — Comparison of RA eQTLs to healthy donor eQTLs. A Comparison of allele frequencies in the RA population used in this study compared to the 1000 genomes dataset for those SNPs that were eQTLs in our dataset. The allele frequency of the peak eQTL for every egene (SNP with lowest p value) was compared to the 1000 genomes project dataset (accessed with Haploreg; http://www.broadinstitute.org/mammals/haploreg/haploreg_v3.php). B Comparison of allele frequencies for all peak eQTLs in our RA population versus the 1000 genomes EUR population. C Comparison of allele frequencies for peak eQTLs that overlap RA GWAS loci or those in high LD (GWAS loci from Okada et al. [2]). D Overlap of egenes in our RA population and the GTEx whole blood dataset (v6). E Comparison of enrichment in RA synovium datasets for egenes that were unique to the RA dataset, the GTEx dataset, or common to both. These datasets used were: RA synovium compared to healthy controls (GSE1919), inflamed RA synovium compared to non-inflamed controls (GSE48780), and RA synovium compared to healthy controls (GSE55235). The p values were generated by a one-sided Fisher’s exact test as described in the manuscript methods. F Selection of enrichment results for the egenes using Nextbio body atlas. The top results for each gene list are shown. The p values are adjusted using the Bonferroni correction. (PDF 7730 kb) [file 13059_2016_948_MOESM13_ESM.pdf]

Figure S9

A

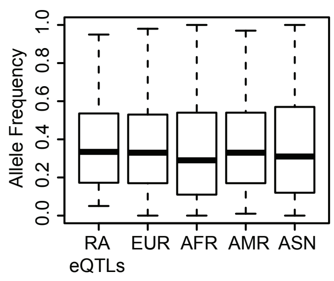

B

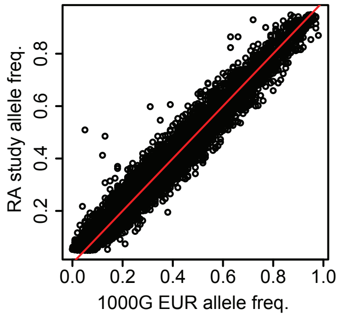

C

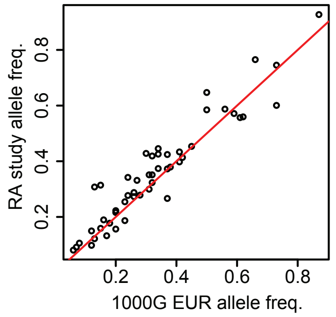

D

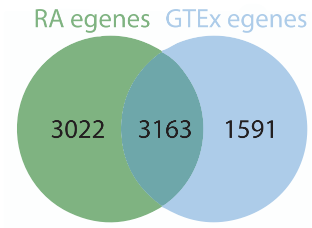

E

NCBI GEO ID	$-\log_{10}$(*p*-value) of enrichment in increased genes			$-\log_{10}$(*p*-value) of enrichment in decreased genes		
	RA *only*	RA *and* GTEx	GTEx *only*	RA *only*	RA *and* GTEx	GTEx *only*
GSE1919	3.1	10.9	0.2	1.4	0.0	0.0
GSE48780	9.5	34.1	1.9	9.7	0.5	0.2
GSE55235	9.2	38.6	2.7	9.1	0.9	0.2

F

Name	RA *only*	RA *and* GTEx	GTEx *only*
B lymphocyte (pre-B) of bone marrow	1.1E-70	4.6E-72	0.20
Macrophage of pulmonary alveolus	8.0E-54	1.6E-69	0.05
Myelocyte of bone marrow	1.7E-50	1.9E-69	0.05
B lymphocyte (immature) of bone marrow	4.1E-59	3.4E-46	0.12
T lymphocyte (CD8+, CD3+) of peripheral blood	2.4E-64	2.0E-41	0.75
T Lymphocyte (regulatory) of peripheral blood	3.1E-59	2.9E-46	0.87
Pro-myelocyte of bone marrow	1.3E-46	2.9E-58	0.46
T lymphocyte (effector) of peripheral blood	7.5E-71	3.1E-35	1.00
Mononuclear cell of peripheral blood	5.3E-49	4.1E-50	1.00
Dendritic cell (immature) of peripheral blood	7.7E-44	4.4E-51	0.37
T Lymphocyte (CD3+) of tonsil	5.4E-62	2.7E-34	1.00
Neutrophil of bone marrow	2.9E-35	8.7E-54	0.71
Leukocyte (polymorphonuclear) of periph. blood	7.5E-40	1.2E-50	1.00
Adipocyte of omentum	1.1E-50	3.4E-31	1.00
Schwann cell of sural nerve	4.4E-30	5.3E-25	0.09
Endothelial cell of lymphatic vessel	1.00	1.00	3.9E-03
T Lymphocyte (CD4+) of tonsil	1.00	1.00	4.4E-03
Hepatocyte	1.00	1.00	4.8E-03
B lymphocyte (naive) of peripheral blood	1.00	1.00	0.02
T Lymphocyte (CD8+) of tonsil	1.00	1.00	0.07
Natural killer cell of tonsil	1.00	1.00	0.10
T Lymphocyte (regulatory) of tonsil	1.00	1.00	0.10
